# Fine-tuned Bee-Flower Coevolutionary State Hidden within Multiple Pollination Interactions

**DOI:** 10.1038/srep03988

**Published:** 2014-02-05

**Authors:** Akira Shimizu, Ikumi Dohzono, Masayoshi Nakaji, Derek A. Roff, Donald G. Miller III, Sara Osato, Takuya Yajima, Shûhei Niitsu, Nozomu Utsugi, Takashi Sugawara, Jin Yoshimura

**Affiliations:** 1 Department of Biological Sciences, Graduate School of Science and Engineering, Tokyo Metropolitan University, Hachioji, Tokyo, 192-0397 Japan; 2 Department of Environmental Sciences, Tokyo Gakugei University, Tokyo, 184-8501 Japan; 3 Department of Biology, University of California, Riverside, CA, 92521 USA; 4 Department of Biological Sciences and Center for Water and the Environment, California State University, Chico, CA, 95929 USA; 5 Department of Zoology, Graduate School of Science, Kyoto University, Kyoto, 606-8502 Japan; 6 Graduate School of Arts and Sciences, University of Tokyo, Japan; 7 Department of Mathematical and Systems Engineering, Shizuoka University, Hamamatsu, 432-8561 Japan; 8 Department of Environmental and Forest Biology, State University of New York College of Environmental Science and Forestry, Syracuse, New York, 13210 USA; 9 Marine Biosystems Research Center, Chiba University, 1 Uchiura, Kamogawa, Chiba-ken, 299-5502 Japan

## Abstract

Relationships between flowers and pollinators are generally considered cases of mutualism since both agents gain benefits. Fine-tuned adaptations are usually found in the form of strict one-to-one coevolution between species. Many insect pollinators are, however, considered generalists, visiting numerous kinds of flowers, and many flower species (angiosperms) are also considered generalists, visited by many insect pollinators. We here describe a fine-tuned coevolutionary state of a flower-visiting bee that collects both nectar and pollen from an early spring flower visited by multiple pollinators. Detailed morphology of the bee proboscis is shown to be finely adjusted to the floral morphology and nectar production of the flower. Behavioral observations also confirm the precision of this mutualism. Our results suggest that a fine-tuned one-to-one coevolutionary state between a flower species and a pollinator species might be common, but frequently overlooked, in multiple flower-pollinator interactions.

Pollination is one of the commonest forms of mutualism between plants and animals^1^,^2^,^3^,^4^. Angiosperms produce flowers with nectar and ample pollen to attract flower-visitors; in return, the flower-visitors transport pollen and effect pollination, so producing seeds. These plant-pollinator interactions are predicted to be the products of coevolution between plants and insects^4^,^5^. A strict one-to-one coevolution is usually found between one plant species and its symbiotic insect partner, e.g., fig plants and fig wasps^6^,^7^. We also find fine-tuned morphological specializations in both particular flowering plants and their specialized insect pollinators^8^,^9^,^10^. However, many angiosperms are visited by myriad pollinators, and many pollinators visit a multitude of flowering plant species. These flowers and pollinators are considered generalists^5^,^11^. The optimal phenotype of a generalist plant is expected to be the intermediate, e.g., the weighted average of all pollinators' phenotypes^12^. Thus we would not expect a fine-tuned specialist in mutualistic interactions between multiple flowers and multiple pollinators.

Contrary to this expectation, we here describe a fine-tuned morphological specialization between an andrenid bee (*Andrena* (*Stenomelissa*) *lonicerae*) and an early spring flower (*Lonicera gracilipes*) visited by multiple pollinators. This flower produces nectar almost exclusively for this bee. We show that the detailed functional morphology of the head and proboscis of the bee is finely adjusted to the morphology and nectar production of the flower. We also demonstrate this fine-tuned specialization from the behavioral repertoire of the bee. We then discuss the implication of this finely tuned one-to-one mutualistic state in the context of the coevolution of pollination interactions between the bee and the flowering plant.

## Results

### Flower visits and nectar

**Figure 1:**
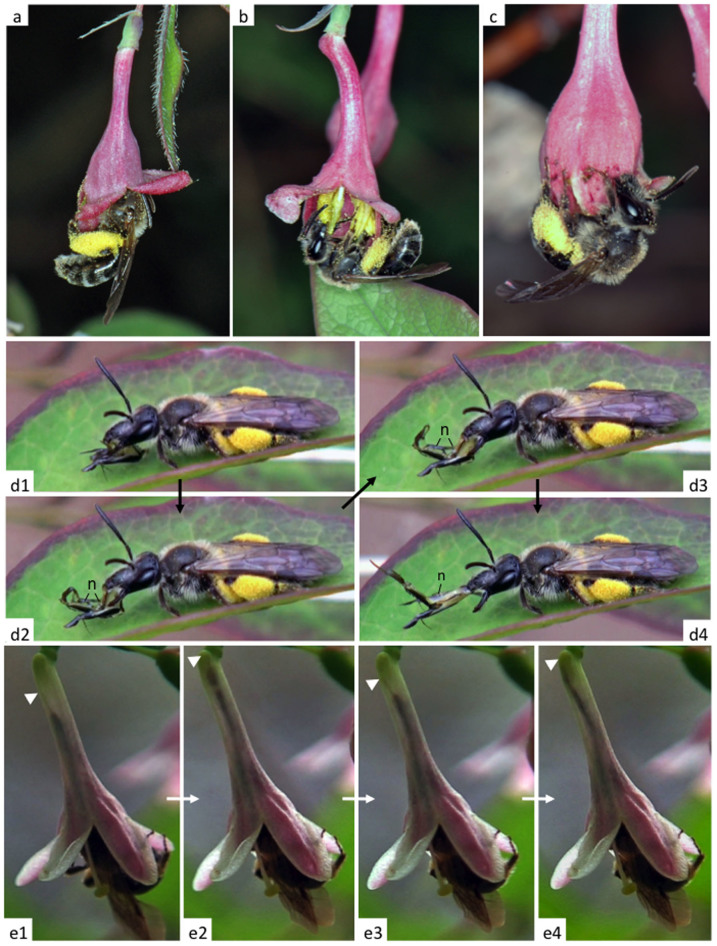
Photos and video pictures of *Andrena lonicerae* bees on flowers of *Lonicera gracilipes*. (a) A female bee taking nectar in the field. (b) A female collecting pollen in the field. (c) A female breaking a bud with her mandibles and fore legs. (d1–d4) Video pictures of extension and retraction of the proboscis in the field. Nectar (n) is seen on the proboscis. (e1–e4) Video pictures of nectar sucking in the field. The tip of the tongue (triangle) extended to the base of the corolla tube (e2 and e4). Collected pollen (yellow) was attached on and near the hind legs (tibiae) (a–d). ((a–c), photographed by X. Fu; others by A. Shimizu).

**Figure 2:**
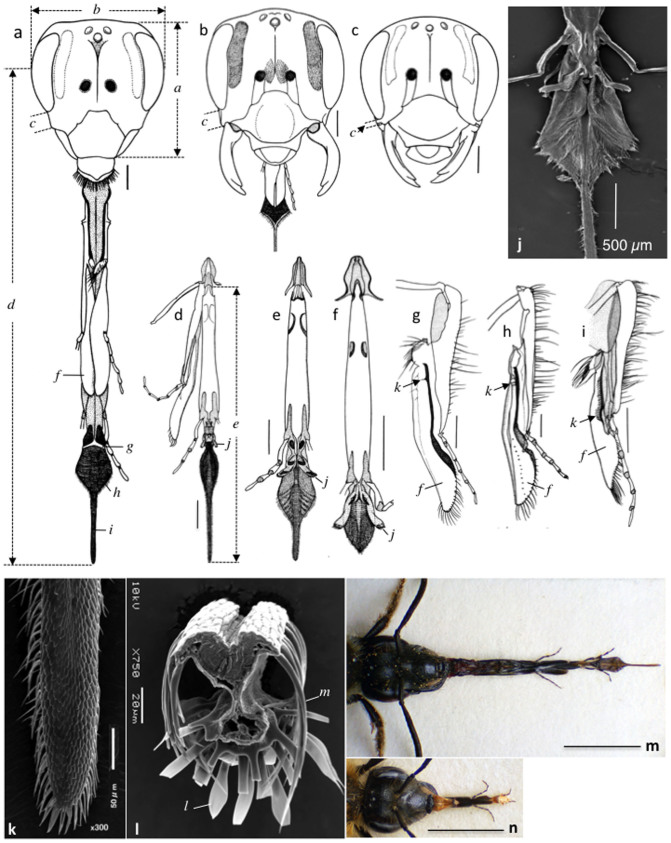
Functional morphology of the head and mouthparts of female *Andrena lonicerae* compared with that of the closely related *A. halictoides* and distantly related *A. hebes* (a–n). (a–c) Head (anterior view): (a) *A. lonicerae* (proboscis fully extended); (b) *A. halictoides* (proboscis partly extended); and (c) *Andrena hebes* (proboscis removed). (d–f) Labium (posterior view): (d) *A. lonicerae*; (e) *A. halictoides*; and (f) *A. hebes*. (g–i) Right maxilla (inner view): (g) *A. lonicerae*; (h) *A. halictoides*; and (i) *A. hebes*. (Scales: 0.5 mm.) (j–m) Ultrastructure of the female *A. lonicerae* glossa: (j) whole glossa, excluding apical part (posterior view); (k) apical part (posterior view); (l) cross section (SEM scan) of apical part (posterior face up); (a-l) Individual parts are (a), head length; (b), head width; (c), malar space length; (d, effective length of head and proboscis for nectar sucking; (e), proboscis length; (f), galea; (g), basiglossal sclerite; (h), main body of glossa; (i), distal part of glossa; (j), paraglossa; (k), galeal comb; (l), annular hair; and (m), seriate hair. (m) Head and fully extended proboscis of *A. lonicerae*. (n) Same of *A. hebes*. Scales: 0.5 mm for (a)–(i); 3.0 mm for (m), (n). (Drawn and photographed by A. Shimizu).

We investigated the pollination activities of females of an andrenid bee, *A. lonicerae*, visiting the flowers of *L. gracilipes* (Fig. 1, Supplementary Videos S1–S3). This andrenid bee is oligolectic (collecting pollen from a limited number of phylogenetically related plants) and is found mostly on *L. gracilipes*^13^,^14^,^15^, though it occasionally visits other flowers (Table 1). *Lonicera gracilipes* blooms in early spring (March to May). Because very few other flowers are available in early spring, *L. gracilipes* is visited frequently by many insects, including another andrenid bee, *Andrena hebes* and halictid bees, *Lasioglossum* spp. (Table 2, Supplementary Fig. S1), although the commonest visitor is *A. lonicerae*. Pollination experiments (Supplementary Fig. S5) reveal that these other visitors may be as equally effective pollinators of this flower as *A. lonicerae*. However, these species collect only pollen, the nectar of this flower being almost exclusively collected by *A. lonicerae* (Table 2). Because the flower of *L. gracilipes* has a characteristic long narrow corolla tube (tube-like shape) (Supplementary Fig. S2), pollinators with a short tongue (mouthparts) have no access to its nectar (Fig. 2, Supplementary Fig. S1, Supplementary Video S5). Remarkably, even though the bee belongs to a group of short-tongued bees (Andrenidae), *A. lonicerae* has an extremely elongate distal part of the tongue (Figs 1d, 2a, d, m, Supplementary Fig. S3a, Supplementary Videos S3, S4). Thus, among all pollinators associated with this flower species, the principal flower-pollinator interaction appears to be that between *A. lonicerae* and *L. gracilipes*.

**Figure 3:**
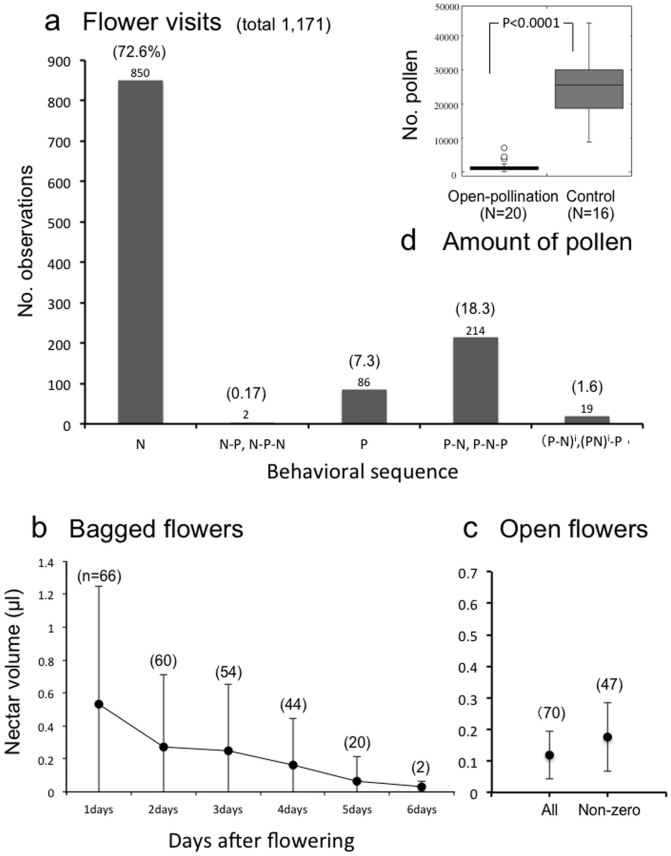
Foraging activity of *Andrena lonicerae* females (a), nectar production of *Lonicera gracilipes* flowers (b), (c), and the number of pollen remaining in flowers (d). (a) Bars indicate the number of observations of various behavior sequences (N: nectar sucking; P: pollen-collection; e.g., N-P: nectar sucking is followed by pollen-collection). The sequence (P-N)^i^ means the repetition of (P-N) for i ≥ 2, such as (P-N-P-N). For example, P-N-P-N-P becomes (P-N)^i^-P (i = 2). P-N-P is grouped in P-N because it could not be discriminated from P-N when a bee started cleaning behavior during or after nectar sucking. The parentheses indicate the relative frequencies (%). (b) Daily changes in the amount of nectar produced by the bagged *L. gracilipes* flowers (Mean ± SD). All fallen flowers were excluded from the measurement (start from 66 sample flowers). Most flowers fell by 5 days. (c) The average amount of nectar found in open flowers (sample size = 70 flowers) including all flowers (left) and excluding 23 flowers with no nectar (right) (Mean ± SD). (d) The number of pollen grains remaining in the anthers of flowers under open-pollination and of flowers in bud (control) (Mean ± SE). Statistical significance was determined by the GLMMs and ANOVA.

Females of *A. lonicerae* visited flowers of *L. gracilipes* to collect nectar (their own energy source) (Fig. 1a) and pollen (their larval food) (Fig. 1b). The foraging activities of the bee indicated that, when pollen was the first target of collection, bees almost always sought pollen before collecting nectar (Fig. 3a). In contrast, when they first took nectar, they rarely looked for pollen (Fig. 3a). There are two probable reasons for this: (1) pollen has already been removed from the flower; and (2) bees are in the pre-nesting stage (no requirement for pollen collection). This asymmetry in behavioral sequence might be explained by the difference between the two resource types: pollen and nectar.

Although a single flower has five anthers (pollen sacks), pollen is a limited non-renewable resource. Because pollen is typically removed by bees in the first couple of days of bloom, the majority of flowers in a blooming period have little pollen (Fig. 3d). When pollen is available, the bees collect it first (Fig. 3a). The limitation of pollen is also suggested by flower-breaking behavior (Fig. 1c): *A. lonicerae* bees sometimes tear off un-opened flower buds by their mandibles and fore legs to collect pollen, nectar or both.

In contrast to pollen, nectar is an extremely limited but renewable resource. During the brief flowering period for each blossom (ca. 4 days), the quantity of nectar secreted daily decreased rapidly from the first (0.53 ± 0.71 μl) to the sixth day (0.03 ± 0.03 μl) (Fig. 3b). Because the amount of nectar per flower is always low (0.12 ± 0.16 μl, n = 70) and many flowers have no nectar (23/70 flowers: 33%) (open flowers; Fig. 3c), the bees must visit the flowers frequently to collect nectar even if pollen is not available (850 visits out of 1171; Fig. 3a). The high frequency of flower-visits of *A. lonicerae* for nectar suggests that *L. gracilipes* provides nectar almost exclusively to this bee.

### Morphology and function of the head and mouthparts

We compared the morphology of the head and mouthparts of *A. lonicerae* females with that of the distantly related *A.* (*Euandrena*) *hebes* and closely related *A.* (*Stenomelissa*) *halictoides* (Fig. 2, Supplementary Fig. S4, Supplementary Table S4). Sympatric and co-occurring *A. hebes* is the second commonest visitor to this flower, but does so only for pollen collection (Table 2). In contrast, *A. halictoides*, mostly allopatric to *A. lonicerae*, is a closely related species that utilizes the funnel-like flower of *Weigela hortensis*, which blooms in mid May^15^.

Among the three species, the female head is extremely elongate in *A. lonicerae*, and considerably so in *A. halictoides*, compared with that of *A. hebes* (Fig. 2, Supplementary Video S5). The length/width proportion of the head is significantly different among the three species, ca. 1.10, 1.00 and 0.92 in *A. lonicerae*, *A. halictoides* and *A. hebes*, respectively (Supplementary Table S4). The oculo-malar space of the head is also considerably elongate in *A. lonicerae* and *A. halictoides* (*c* in Fig. 2a and b) compared with the more typical short malar space of *A. hebes* (*c* in Fig. 2c). The proportion of the malar space to the head width is also significantly different among species, ca. 0.12, 0.09 and 0.04 in *A. lonicerae*, *A. halictoides* and *A. hebes*, respectively (Supplementary Table S4). This suggests that the elongation of the head of *A. lonicerae* is an adaptation to tube-shaped flowers, such as those of *L. gracilipes*, the floral morphology of which is described below; the same applies to *A. halictoides* on *W. hortensis*^15^.

The mouthparts of both *A. lonicerae* and *A. halictoides* females also exhibit distinctive specializations (Fig. 2). The distal part of the tongue (glossa) is greatly extended in *A. lonicerae* (*i* in Fig. 2a; Fig. 2d, m), resembling a string, and fairly elongate in *A. halictoides* (Fig. 2b, e), compared with that of *A. hebes* (Fig. 2f, n). We should note that, among approximately 1,500 species of the genus *Andrena*, *A. lonicerae* and *A. halictoides* are the only two species possessing an elongate distal portion of the glossa, hence their placement in the subgenus *Stenomelissa*. The paraglossa covering the basal part of the glossa laterally is rudimentary in both *A. lonicerae* and *A. halictoides* (*j* in Fig. 2d and e, *b* in Supplementary Fig. S4c compared with *j* in Fig. 2f, and *b* in Supplementary Fig. S4b).

In both *A. lonicerae* and *A. halictoides*, there is also a distinctive modification in the maxillary galea covering the basal proboscis dorsally (*f* in Fig. 2a). The brush-like structure called the galeal comb (cleaning apparatus located on the inner face of the galea) shared with almost all short-tongue bees has undergone a reduction to one to three bristles (setae) (*k* in Fig. 2g and h, cf. *k* in Fig. 2i, Supplementary Table S5). The loss of almost all setae in the galeal comb and the considerable reduction in the size of the paraglossa are unique features found elsewhere in the long-tongued bees^16^,^17^, but are atypical of short-tongued bees, indicating convergence between long-tongued bees and *A. lonicerae* and *A. halictoides*.

In addition to the gross anatomy of the glossa of *A. lonicerae*, we examined its ultrastructure (basal part: Fig. 2j; distal parts: Fig. 2k, l). The anterior surface of the distal portion is covered with long hair-like cuticular processes (annular hairs) (*l* in Fig. 2l) as in the basal part of the glossa (*h* in Supplementary Fig. S4j), while its posterior portion has a single row of arched spine-like cuticular processes (seriate hairs) on both sides (*m* in Fig. 2l). The embedded cross section of this portion reveals that its posterior is a dense matrix of tissue, while the anterior contains mostly body fluid (Fig. 2l, Supplementary Fig. S4k, m), allowing flexibility. The row of cuticular processes along the lateral margins of the distal glossa of *A. lonicerae* and *A. halictoides*, which forms a tunnel-like space suitable for holding nectar, is distinctly different from the capillary (hairy) structure of the long-tongued bees, where the glossa is covered with transverse rows of slightly flattened, similar cuticular processes both anteriorly and posteriorly (Supplementary Fig. S4n–q). The elongation of the distal glossa, prementum, and malar space, together with the narrow head of the bee (Fig. 2a, d, Supplementary Table S4), are thus finely tuned for extracting nectar from the base of the corolla tube (Fig. 1a, e, Supplementary Fig. S3a, Supplementary Videos S1, S3).

### Molecular phylogeny of bees

We examined the molecular phylogenetic relationship among *A. lonicerae*, its morphological sister species *A. halictoides*^14^ and other *Andrena* bees, using the maximum likelihood method and Bayesian method. We confirmed that *A. lonicerae* and *A. halictoides* are monophyletic sister species distantly related to all other examined *Andrena* species (Fig. 5, Supplementary Fig. S6). The sequence data also indicated that *A. lonicerae* is derived from the ancestral *A. halictoides*. We also verified the current results using the neighbor joining and maximum parsimony analyses.

### Floral morphology and behavioral verification

**Figure 4:**
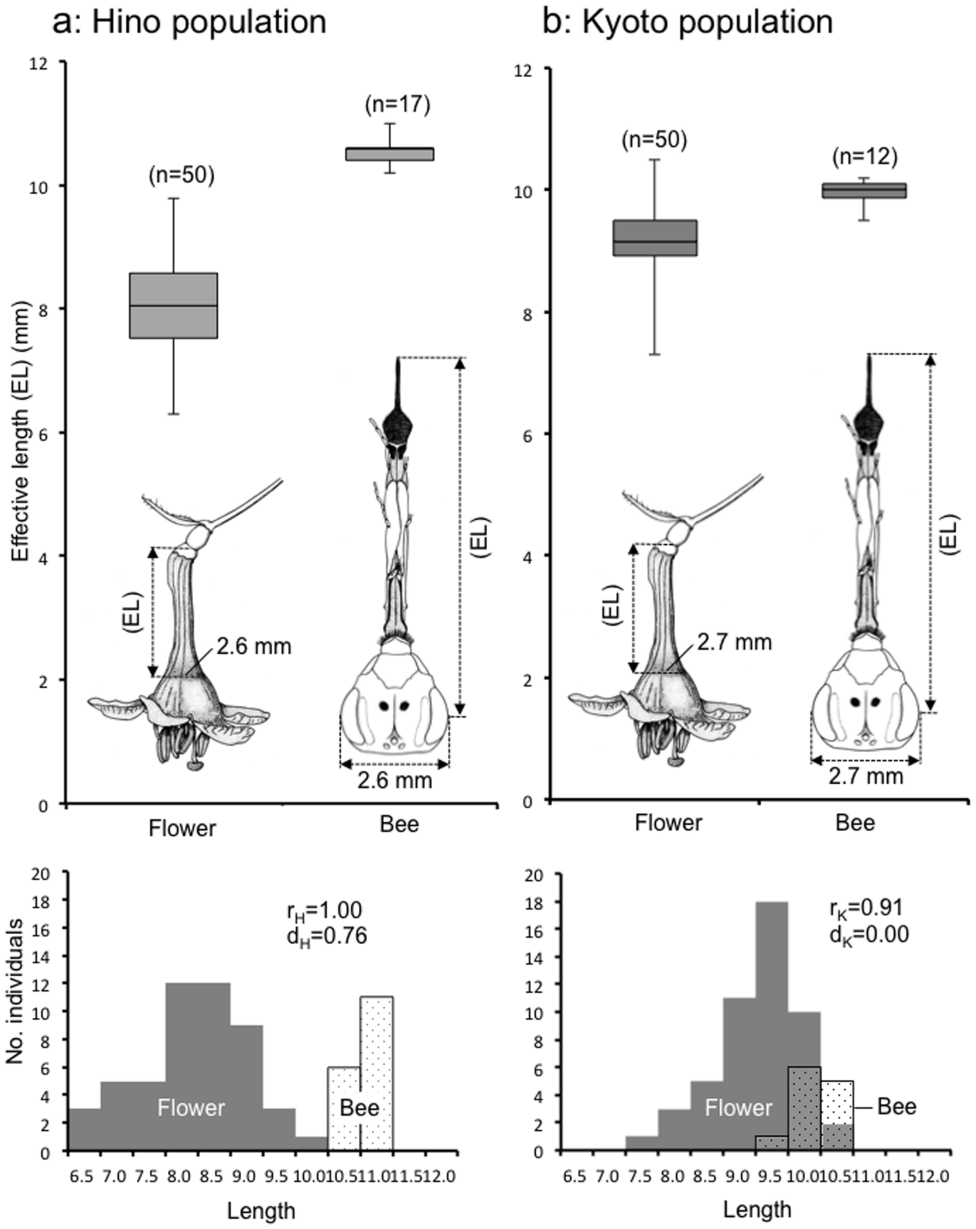
Comparison of the effective length (EL) between the corolla tube and the nectar-sucking part of the bee. (a) Hino population. (b) Kyoto population. Upper column: the EL (median, upper/lower quartiles, maximum and minimum) of both the corolla tube and the bee head parts (n: sample size). Lower Column: length distribution of both the corolla tube and the head parts. The EL of the corolla tube is determined by the opening width of a flower that corresponds to the head width of the bee at each locality (Hino: 2.6 mm; Kyoto: 2.7 mm). The bees' EL and flowers' EL are 10.6 ± 0.21 mm and 8.0 ± 0.83 mm, respectively in the Hino population; and 10.0 ± 0.22 mm and 9.2 ± 0.61 mm, respectively in the Kyoto population. The nectar-reaching rate *r* (unit: %) and the departure index *d* (unit: mm) are shown for each population (definition and calculation of r and d, see Supplementary Information).

Detailed measurements of *A. lonicerae* reveal a precise correspondence with the tube-like flower of *L. gracilipes* (Fig. 4). Because nectar quantities tend to be very small, the bee has to extend its proboscis fully to collect any available nectar at the base of the funnel-shaped flower (Fig. 1e). We found extremely good agreement between the effective length (EL) of the bee's head and proboscis (see “MATERIALS AND METHODS” in Supplementary Information) and that of the corolla tube in both the Hino and Kyoto populations (Fig. 4). From the nectar-reaching rate r, all bees (100%) have an access to nectar in all flowers in the Hino population, and 91% of bees in the Kyoto population. At the same time, the departure index is 0.76 mm in the Hino population and 0.00 mm in the Kyoto population. These two measures indicate the very close match between the proboscis length and corolla tube length in both the Hino and Kyoto populations.

Using video recording, we investigated the ability of the bee to reach nectar at the base of the flower (Fig. 1e, Supplementary Video S1). The bee puts its head deep into the flower (corolla tube) when it searches for nectar (Fig. 1a, e), whereas it hangs on the flower without inserting its head into the corolla when collecting pollen from anthers (Fig. 1b, Supplementary Video S2). When sucking nectar, the bee extends its mouthparts within the corolla tube (Supplementary Fig. S3a, Supplementary Videos S1, S3). The proboscis, which is folded three times under the head, is highly flexible and fully extended when necessary (Fig. 1d, Supplementary Video S4). The motion picture silhouettes of a bee sucking nectar show that the apex of the glossa reaches the base of the flower (Fig. 1e, Supplementary Video S1). These results demonstrate a fine one-to-one morphological match between the bee's proboscis and the corolla tube. Such morphological specializations, together with the behavioral data that more than 70% of flower visits are solely for obtaining nectar (Fig. 3a), indicate that *A. lonicerae* is mostly dependent on *L. gracilipes* as its source of nectar.

Thus both morphological and behavioral adaptations of the bee, as well as nectar production by the flower, indicate that this flower-pollinator interaction is highly mutualistic. This tight mutualism is strongly supported by the close matching between the effective length of the head and mouthparts of the bee, and that of the corolla tube, in two separate populations (Fig. 4). The behavioral video recordings demonstrate a fine-tuned nectar collecting behavior (Fig. 1e, Supplementary Video S1).

## Discussion

In most pollination systems, flowering plants are typically visited by many pollinator species, while a given pollinator often visits many different flower species^2^,^3^. In such cases both flowers and pollinators are often considered generalists^12^,^18^. The bee *A. lonicerae* visits mostly the flower *L. gracilipes* and, rarely, other flowers, exhibiting narrow oligolecty^14^,^19^ (Table 1). This vernal flower otherwise appears as a generalist in that it is visited by many different insects (at least 11 species; Table 2). Hence the relationship examined here embodies an interaction between an oligolectic bee and a generalist flower. The close matching of the head and mouthparts of the bee to the tube length of the flower indicates that both the flower and the bee might have coevolved (i.e., have responded to one another evolutionarily) (Fig. 4).

Our data (Supplementary Fig. S5) show that the pollination effectiveness of one visit by other frequently visiting bees to *L. gracilipes* (e.g., *A. hebes*) is almost equivalent to that of *A. lonicerae*. However, assuming these bees constitute evolutionary partners (as effective pollinators) of *L. gracilipes*, its nectar would be expected to be accessible to these bees. The bees, however, cannot gain access to the nectar because their mouthparts are too short to reach the base of the corolla tube (Fig. 2n, Supplementary Fig. S1, Supplementary Video S5). This indicates that they are only riders, i.e., free loaders. The following preliminary observations provide support: *A. lonicerae* frequently displayed multiple visits from flower to flower for nectar (see also Fig. 3a), whereas the other visiting bees tended to abandon a patch of flowers after a single visit to a flower for pollen.

Close examination of the interaction between *A. lonicerae* and *L. gracilipes* reveals a tight one-to-one relationship. Among all visitors to *L. gracilipes*, *A. lonicerae* is the most frequent, and essentially the only one to collect both nectar and pollen (Table 2). Although nectar production is very small in this flower (on average 0.12 μl/open flower in Fig. 3c), 72.6% of the bee's visits are solely for nectar (Fig. 3a). These data indicate that the flower is the main nectar source for the bee. Thus the present bee-flower relationship differs considerably from a generalist pollination system, even though multiple pollinators visit *L. gracilipes* flowers, and *A. lonicerae* bees occasionally visit other flowers.

**Figure 5:**
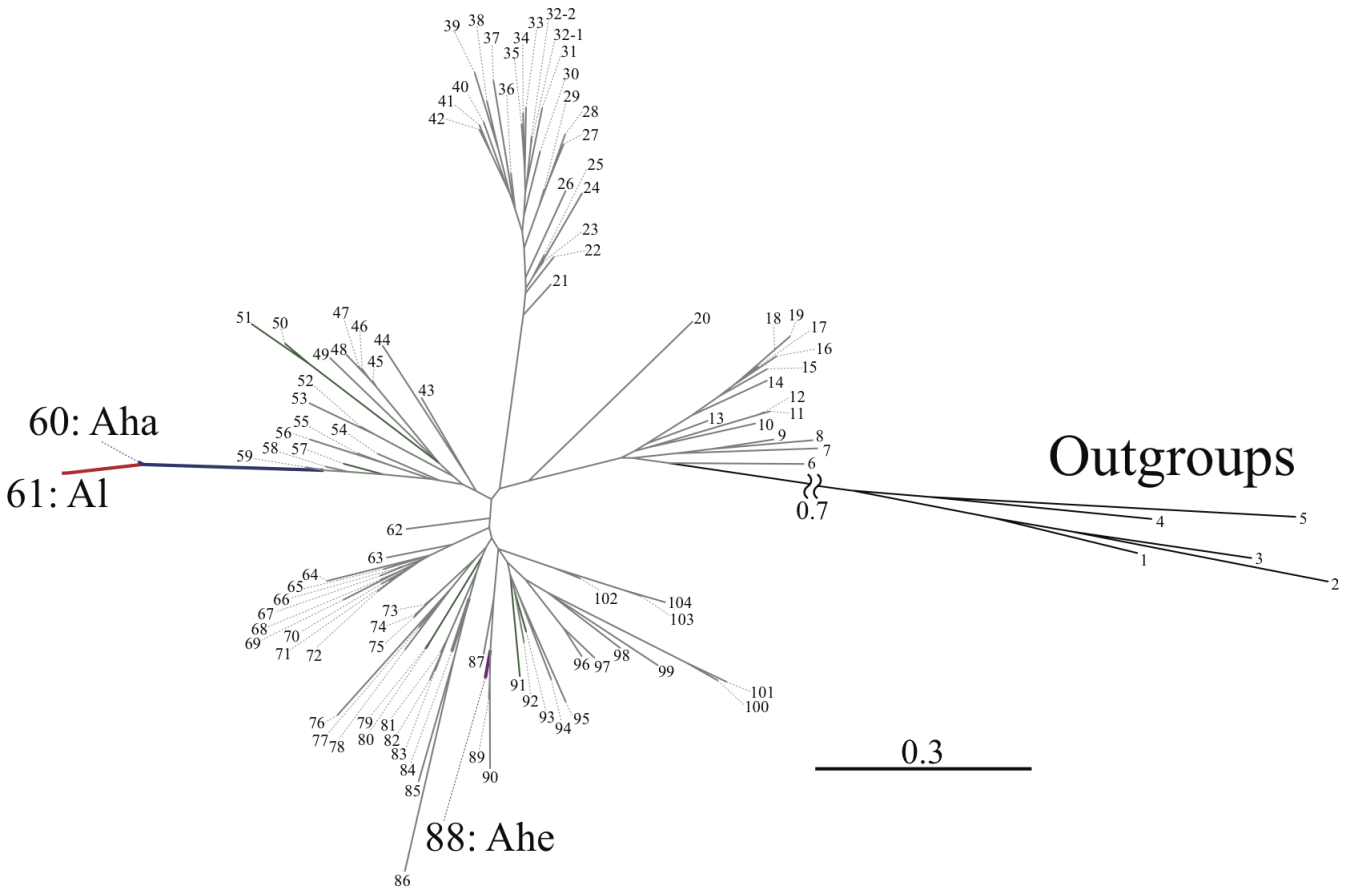
Molecular phylogenetic relationships of the genus *Andrena* based on the mitochondrial COI, tRNA-Leu and COII regions (without third codons) using maximum likelihood method with special reference to Japanese species. The tree contains the *A. lonicerae* clade (Al: red branch), *A. halictoides* clade (Aha: dark blue branch), *A. hebes* clade (Ahe: dark violet branch), other Japanese *Andrena* species (dark green branch) and non-Japanese species (light grey branch). The operational taxonomic units (species) except the above three Japanese species were indicated in numbers. Species names were shown in Supplementary Table S2.

The phylogenetic background of *A. lonicerae* also supports this fine-tuned specialization between the bee and the flower. Among the approximately 1,500 species of *Andrena* classified as short-tongued bees, all are known to have a short tongue except four species. Among these exceptions, two species, *A.* (*Iomelissa*) *violae* and *A.* (*Callandrena*) *micheneriana*, occur in the USA^17^,^20^,^21^,^22^, and are not closely related to the other two species, *A.* (*Stenomelissa*) *halictoides*, which occurs in China, Korea, Japan and Russian Far East, and *A.* (*S.*) *lonicerae*, which is endemic to Japan^14^,^23^. From morphological specializations of the head and mouthparts (malar space, glossa, paraglossa and galeal comb in Fig. 2a, b, d, e, g, h, m), the two East Asian species, belonging to the subgenus *Stenomelissa*, are definitely closely related sister species, distinct from other species of *Andrena*. The current molecular analyses support the monophyly of the two species (Fig. 5, Supplementary Fig. S6). The slightly elongate, string-like distal glossal portion of *A. halictoides* (Fig. 2b, e) has been further elongated in *A. lonicerae* (Fig. 2a, d, m). The elongation of the mouthparts of *A. lonicerae* is also reflected in the total length of the proboscis (Supplementary Table S4). From both morphological specialization and molecular phylogenetic data, *A. lonicerae* appears to be derived from the ancestral *A. halictoides* (Fig. 5, Supplementary Fig. S6).

The geographic distributions of both the bees and their floral hosts also support this evolutionary scenario. Most *Andrena* bees exhibit oligolectic (larval) pollen diets, exhibiting fairly tight relationships with their host flowers^24^. In Japan, nectar diet specialization is also found in the two *Andrena* bee species^14^,^15^: *Andrena halictoides* is fairly oligolectic on *Weigela hortensis*, but is rarely seen on many different flowers, while *A. lonicerae* is more (but not strictly) oligolectic on *L. gracilipes*. These two bee species, together with their corresponding flowering plant partners, exhibit a nearly allopatric distribution, with only narrow overlapping regions^15^,^25^. *Andrena* *halictoides* and its host plant *W. hortensis* occur on the cooler Japan Sea side of Honshu and Hokkaido. Although the bee is also distributed in the eastern parts of the Asian Continent, the distributions of the bee and its host plant are almost coincident with each other in Japan. In contrast, *A.* *lonicerae* and its host *L. gracilipes* occur on the warmer Pacific Ocean side of Honshu, and their distributions coincide nearly perfectly. The flowering period of *L. gracilipes* is early spring (March–May) on the Pacific Ocean side, while that of *W. hortensis* is late spring (May–June) on the Japan Sea side. The flowering periods of the two species may be less distinct or even overlap in northern and mountainous Honshu, where both plant species may occur in the same locations. In fact, there is a record of *A. lonicerae* visiting flowers of *W. hortensis* (Table 1). Hayashibara *et al.*^15^ found that some females of *A. lonicerae* visit flowers of *W. hortensis* to forage for pollen after the flowering period of *L. gracilipes*. We thus suggest that *A. lonicerae* originated from an ancestral population of *A. halictoides* in the region where *W. hortensis* and *L. gracilipes* overlapped, undergoing a shift from *Weigela* to *Lonicera* and spreading its distribution from the Japan Sea side of Honshu to the Pacific side.

Based on their molecular phylogenetic studies of *Lonicera*, Theis *et al.*^26^ suspected that *L. caerulea* is the sister species of *L. gracilipes*, although they included only eight of the 21 Japanese species in their analyses (note that the species name *L. caelurea* is misspelled as *L. coerula* in the tree of Fig. 1 and as *L. coerulea* in Appendix 1). *Lonicera caerulea* is widely distributed in the cool-temperate and temperate Northern Hemisphere from Europe through Japan to North America, with several named subspecies. *Lonicera caerulea* subsp. *edulis* is distributed from eastern Siberia to the Korean peninsula, as well as in Hokkaido and the subalpine zone of central and northern Honshu^27^. In contrast, *L. gracilipes* is endemic to Japan, ocurring mostly in the mountainous regions of the southeastern regions of Japan^27^. The two species are thus allopatrically distributed.

Judging from the wide distribution of *L. caerulea* subsp. *edulis* and the peripheral distribution of *L. gracilipes* in warmer climatic zones, the latter is suspected to have evolved from the former. The phenological and morphological specializations of *L. gracilipes* also suggest that *L. gracilipes* is likely to have evolved from the ancestral lineage of *L. caerulea*. The two flowering plants are distinct in their phenology and morphology: *L. gracilipes* blooms in March to April, while *L. caerulea* subsp. *edulis* blooms in May to July. The tubular part of the corolla of *L. gracilipes* is 10–12 mm, which is almost unique among the Japanese species of *Lonicera*, while that of *L. caerulea* subsp. *edulis* is 5–8 mm in (Table 3).

According to Kato^28^, *Lonicera* is a taxon having coevolved with bumblebees, although the three Japanese species, *L. japonica*, *L. hypoglauca*, and *L. affinis* (all with very long corolla tubes) are pollinated by hawk moths. Similarly, some North American *Lonicera* species with elongated red corolla tubes are pollinated by hummingbirds. Kato presumed that, excepting the above three species, the effective pollinators of all Japanese *Lonicera* are bumblebees, typifying the insect visitors to eight *Lonicera* species.

From the above discussions, we suspect that *L. caerulea* subsp. *edulis* first had expanded its range to southeastern Japan, adapting to the warmer, sunnier and much less snowy winter climate of that found currently in southeastern Japan. One important aspect of this complex history is a suspected pollinator shift from bumblebees to *A. lonicerae* or *A. halictoides*. Two floral traits are characteristic of *L. gracilipes*: 1) the corolla tube length is much longer in *L. gracilipes* than in *L. caerulea* sub. *edulis* (Table 3); 2) the quantity of nectar provided by *L. gracilipes* is much lower than that of many flowering species typically visited by bumblebees. For example, nectar production of *Weigela coraeensis*, which is utilized mostly by two species of bumblebees (*Bombus ardens* and *B. diversus*) is 2.2 ± 3.0 μl/bagged flower/day (mean ± SD, n = 304; original data) in volume. In contrast, nectar production of *L. gracilipes* is 0.3 ± 0.51 μl/bagged flower/day (mean ± SD, n = 246). Hence ancestral *L. gracilipes* populations may have undergone an elongation of the corolla tube and a reduction in nectar output during their evolution from the ancestral *L. caerulea* stock. Meanwhile, *L. gracilipes* presumably underwent a shift from an unknown pollinator (bumblebees or the ancestral *A. halictoides*) to *A. lonicerae*. Elucidating this process of pollinator shifting at the origins of *L. gracilipes* requires identifying the pollination syndrome and current pollinators of *L. caerulea*. We conclude that our finding of a one-to-one matching between *A.* *lonicerae* and *L. gracilipes* demonstrates a high probability that coevolution has occurred between them.

Coevolution in pollination is often considered in the context of specialists and generalists, where both pollinators and flower specialists have evolved from generalists and *vice versa*^4^,^5^. The reason why we cannot identify the mutual partner easily is that there may be many so-called rider species (e.g., free loaders, lobbers/thieves) that utilize or steal pollen and nectar from the flower. A possible exception is social bees that collect nectar and pollen from available flowers in their surrounding environment for a long period^19^. As with *L. gracilipes*, many apparently generalist flowers may be in fact finely tuned to a single pollinator. The oligolectic pollen diet of *Andrena* bees also constitutes evidence for this narrow specialization^24^. In almost all symbiotic and mutualistic systems, we may find species-specific one-to-one interactions.

Unlike many other mutualistic (symbiotic) phenomena typically exhibiting a one-to-one relationship, pollination has been widely considered to consist of multiple interactions between many pollinators and many flowers (e.g., wild flower gardens in highlands and mast flowering in tropical rain forests^4^). Only a few special cases of one-to-one pollination interactions are known, e.g., fig wasps and fig trees^6^,^7^ and some flowers with long nectar spurs and their specialized pollinators (bumblebees, hawk moths and hummingbirds)^9^. These are exclusive interactions involving no other pollinators, nor other flowering plants. Our findings suggest a possible mutual interdependence, but not necessarily a strictly exclusive interaction. In the context of a broad coevolutionary spectrum ranging from multiple interactions to one-to-one exclusive interactions in pollination systems, we may expect many non-exclusive one-to-one interactions and their intermediate ones hidden within superficially multiple interactions. Considering the stability of pollination systems, one-to-one interactions are likely to be favorable, as opposed to the one-to-many or many-to-many interactions, typical in other mutualisms.

With respect to pollination ecology, our findings imply that co-adaptation (coevolution) and mutual relationships (interactions) are two different things. Multiple mutual interactions do not mean multiple co-adaptations. Many angiosperms may have a target pollinator that guarantees the pollination of their own flowers. Many insect pollinators with only a short active period may also have a target plant species when they search for flowers, as does *A. lonicerae*. Hence one-to-one co-adaptation might have evolved and been retained within multiple pollination interactions.

## Methods

We here describe a fine-tuned morphological specialization between an andrenid bee (*Andrena* (*Stenomelissa*) *lonicerae*) and an early spring flower (*Lonicera gracilipes*) visited by multiple pollinators. This flower produces nectar almost exclusively for this bee. We show that the detailed functional morphology of the head and proboscis of the bee is finely adjusted to the floral morphology and nectar production of the flower. We also demonstrate this fine-tuned specialization from the behavioral repertoire of the bee. See Supplementary Information for the detailed methods.

## Author Contributions

A.S. and J.Y. conceived the study. A.S., S.O., T.Y., S.N., M.N., I.D. and T.S. collected samples and field data, M.N. and N.U. performed molecular analysis, A.S., I.D., D.R., D.M. and J.Y. conducted data analyses, D.S. and A.S. performed statistical analyses. A.S., J.Y., D.R. and D.M. wrote the manuscript.

## Supplementary Material

Supplementary Information Supplementary Information

Supplementary Information Supplementary Video S1.

Supplementary Information Supplementary Video S2.

Supplementary Information Supplementary Video S3.

Supplementary Information Supplementary Video S4.

Supplementary Information Supplementary Video S5.

Supplementary Information Supplementary Video S6.

## Figures and Tables

**Table 1:** Flower records of *Andrena lonicerae* (Tadauchi & Hirashima 1988)

	No. of specimens collected	
Plant family/species	Female	Male
Caprifoliaceae		
*Lonicera gracilipes*	33	5
*Weigela hortensis*		1
Ericaceae		
*Rhododendron metternichii*	1	
Elaeagnaceae		
*Elaeagnnus* sp.	1	
Aceraceae		
*Acer* sp.		1
Rosaceae		
*Malus sieboldii*		1

**Table 2:** The number of insect visitors to flowers of *Lonicera gracilipes* at Hino study site. Observation dates are April 1, 2, 4, 5, 6–10, 12, 13, 2012. Each observation time (hrs.) is indicated in parentheses. The data of two populations are combined (see also Supplementary Fig. S1)

Insect visitors*1	Floral resource	Total (32.6)	1 Apr (1.7)	2 Apr (2.3)	4 Apr (2.2)	5 Apr (3.0)	6 Apr (3.3)	7 Apr (3.4)	8 Apr (3.0)	9 Apr (3.2)	10 Apr (3.9)	12 Apr (3.8)	13 Apr (2.8)
*Andrena* (*S.*) *lonicerae*, female	pollen, nectar	255	4	6	7	9	27	14	38	54	25	42	29
*A.* (*S.*) *lonicerae*, male	nectar	19	1	2	3	4	2	0	2	0	3	1	1
*Andrena* (*E.*) *hebes*, female	pollen*2	218	28	48	32	26	6	8	32	10	22	4	2
*Lasioglossum* spp., female	pollen	132	1	1	2	23			1	43	33	20	8
*Ceratina japonica*, female & male	pollen*3, nectar	30				12				6	9	3	
*Eucera nipponensis*, female	nectar	15									2	4	9
*Nomada* sp.	nectar	3								1		1	1
*Osmia* spp., male	nectar	10							1	1	4	3	1
*Bombus ardens*, queen	nectar	1				1							
*Bombylius major*	nectar	42		1	14	8	1	10	6	2			
Syrphid flies	pollen	2							1	1			
*Erynnis montanus*	nectar	9		2	7								

*1, In Hymenoptera, *A.* (*Stenomelissa*) *lonicerae* and *A.* (*Euandrena*) *hebes* belong to the family Andrenidae, and *Lasioglossum* spp. (either *L. vulsum* or *L. proximatum*), to Halictidae. Other bees (Hymenoptera) include the families Megachilidae (*Osmia* spp.: either *O. cornifrons* or *O. taurus*). Apidae (*Bombus ardens*), and Anthophoridae (*Ceratina japonica*, *Nomada* sp., *Eucera nipponensis*). Other insects are two species of Diptera (Bombyliidae: *Bombylius major* and Syrphidae: unidentified sp.) and one species of Lepidoptera (Hesperiidae: *Erynnis montanus*).

*2, A few individuals appeared to try to take nectar by entering the corolla tube deep.

*3, Only females forage for pollen.

**Table 3:** Flower length of the Japanese species of *Lonicera* (based on Hara^25^)

Species	Length of corolla (mm)	Length of tubular part of corolla (mm)
*L. japonica*	25–35	14–22
*L. hypoglauca*	40–50	25–30
*L. affinis*	30–45(50)	15–20
*L. maackii*	17–22	5
*L. morrowii*	–––*	4–5
*L. demissa*	8–13	3–3.5
*L. chrysantha*	12–15	4–5
*L. caerulea*	10–15	5–8
*L. gracilipes*	12–20	10–12
*L. linderifolia*	7–9	5
*L. strophiophora*	20–25	13–15
*L. praeflorens*	–––*	4–5
*L. cerasina*	6–12	3–5
*L. vidalii*	–––*	5–7
*L. alpigena*	11–14	4–5
*L. ramosissima*	15–20	11–15
*L. chamissoi*	8–12	3
*L. maximowiczii*	6–9	4
*L. mochidzukiana*	10–13	4
*L. tschonoskii*	12–15	4
*L. harai*	10	4–5

*No data probably because of the corolla lobe arranged radically.
