# Supplementary material for: Fine-tuned Bee-Flower Coevolutionary State Hidden within Multiple Pollination Interactions
Source: Sci Rep. 2014 Feb 5;4:3988. doi: 10.1038/srep03988 (PMC3913927; doi:10.1038/srep03988)
Supplement: Supplementary Information [file srep03988-s1.pdf]

Fine-tuned Bee-Flower Coevolutionary State Hidden within Multiple Pollination Interactions

Akira Shimizu, Ikumi Dohzono, Masayoshi Nakaji, Derek A. Roff, Donald G. Miller III, Sara Osato, Takuya Yajima, Shûhei Niitsu, Nozomu Utsugi, Takashi Sugawara and Jin Yoshimura

Supplementary Information

SI Text

MATERIALS AND METHODS

Bees examined

Andrena is the largest genus in the Andrenidae (Apoidea) comprising as many as 1,500 described species in 96 subgenera, and is predominantly Holarctic[17,29]. This genus is one of the 'short-tongued bee' groups and includes both polylectic and oligolectic taxa[30–32]. For this reason, it is an excellent group for investigating coevolution of bees and flowers in terms of oligolecty and polylecty[24].

The subgenus *Stenomelissa* of the genus *Andrena*, to which *A. lonicerae* Tadauchi and Hirashima, 1988 and *A. halictoides* Smith, 1869 belong, is endemic to East Asia. This subgenus includes another species, *Andrena vitiosa* Smith, 1879 occurring in China[14,23], although its subgeneric position is questionable to us (known only from the holotype male and another male specimen). *Andrena lonicerae* and *A. halictoides* are nearly allopatric in Japan, i.e., *A. lonicerae* is distributed in Kyushu, Shikoku, and the Pacific side of Honshu, whereas *A. halictoides* is distributed in Hokkaido and mountainous regions and the Japan Sea side of Honshu, as well as northeastern China, Korea, and Primorsky Kray, Russian Far East[14,23,25]. They are narrowly sympatric in the Sanin district (Japan Sea side of Western Honshu) and central Tohoku district, Honshu[15,25].

Andrena lonicerae is known to be an oligolege (specialist pollinator) of *Lonicera gracilipes* Miq., and *A. halictoides*, of *Weigela hortensis* (Siebold et Zucc.) Koch (both Caprifoliaceae) [14,15,33]. These bees have an elongate head and proboscis adapted for collecting nectar from tube-shaped flowers[13,15,33]. Hayashibara *et al.*[15] studied the pollination syndrome of the two flower species, *L. gracilipes* and *W. hortensis* in relation to the two bee species in the Sanin district, where both bees and plants are sympatric. They found phenological and morphological matching between the flowers of the plants and the corresponding oligolectic bees, and concluded that *A. lonicerae* is the sole effective pollinator of *L. gracilipes*, whereas *A. halictoides* is the primary pollinator of *W. hortensis*. However, detailed matching among head-proboscis morphology and foraging behavior of *A. lonicerae* and floral morphology of *L. gracilipes* have not yet been examined. In our analyses, male bees were excluded, since their head size shows great variation, possibly owing to severe mating competition among males within communal nests.

Host plant

Lonicera gracilipes is a deciduous shrub at most 3 m in height, usually in the understory of broad-leaved forests or on their edges. This species is distributed widely in Japan from Kyushu and Shikoku throughout the Pacific Ocean side of Honshu to southern Hokkaido[25,27]. On the Kanto plains, including Tokyo, it blooms from early- or mid-March to mid- or late April. *Andrena lonicerae* is univoltine and active chiefly from mid-March to early May. The flowering period of *L. gracilipes* is nearly synchronous with the bee's flight season. Therefore, the mutually interdependent pollination interaction between them occurs but once a year. We observed the interaction at two populations in Hino city, as mentioned below.

Field investigation on insect visitors to the flowers

Insect visitors to flowers of *L. gracilipes* were recorded from about 9:30 to 14:00 on 1, 2, 4–10, 12, and 13 Apr 2012 (a total of 36.2 hours) at two populations in Hino City, Tama Hill, Tokyo, Japan, where several populations of *L. gracilipes* occur at the edges of temperate deciduous forests dominated by *Quercus serrata* Murray. Walking slowly and continuously around stands of the host plants, we counted the number of insects foraging on the flowers. Inspecting all blossoms in each 10–20 m^2 stand required three to five minutes.

Because of the ambient climatic conditions prevailing in spring, we were limited to observing insect visitors only during the late morning and early afternoon. In Hachioji city, which is adjacent to Hino city, the average, maximum, and minimum temperatures of the last ten days of March and the first ten and middle ten days of April in 1983–2010 are shown in Supplementary Table S1. In general, the minimum temperature is recorded at daybreak, and nighttime temperatures range from 4–8 °C in late March and early April. Such temperatures are prohibitively low for most insect activity. At our study site, we observed no nocturnal insect activity during this early spring season. In the mid to late afternoon, it often became windy (spring winds are mostly northerly winds) and cloudy, and the surface temperature went down quickly, at which time all flower visits quickly ceased.

We observed foraging behavior of bees on the flowers in the spring of 2007–2013, at the same plant populations. The total observation time was 152.5 hours over 63 days.

The behavior of the bees was recorded with video cameras and was afterward analyzed using these recordings.

Morphological investigation on the head and mouthparts of bees

To investigate the morphology of the mouthparts, the labio-maxillary complex was detached from the cranium of bee specimens that were softened with steam beforehand. The labium was then observed under a stereoscopic microscope, and was photographed with a digital camera. For scanning electron microscopy study, the air-dried labium was mounted on a specimen stub, and was coated with gold to a thickness of ca. 20nm in a JEOL JFC-1100E ion sputter. The microscope used was a JEOL JSM-5610LV, at a working voltage of 10kV.

To observe the cross section of the glossa, the tissues were fixed in Karnovsky's fixative (2% paraformaldehyde + 2.5% glutaraldehyde) and 1% osmium tetroxide. After dehydration through a series of ethanol and propylene oxide solutions, the tissues were embedded in Epon 812 (TAAB). Semi-thin sections 1µm thick were made on a rotary microtome, mounted on microscopic slides, stained with Azur-B, and then observed with an optical microscope.

The terminology of the glossa follows Michener and Brooks[34] and Michener[17], and that of the postmentum, Plant and Paulus[35]. The following five morphological characters of *A. lonicerae* and *A. halictoides* were measured with a micrometer scale: (1) head length (measured from the apical margin of the clypeus to the crest of the vertex) (Fig. 2a: *a*); (2) head width (Fig. 2a: *b*); (3) malar space length (Fig. 2a: *c*); (4) proboscis (prementum + glossa) length (Fig. 2d: *e*); and (5) length of the head and proboscis taken together (Fig. 2a: *d*). For the last metric, the labio-maxillary complex of fresh specimens was fully extended forward before the head was glued to cardboard (Supplementary Fig. S1), then the length from the glossal tip to the point on the midline of the head at the maximum head width was measured as the effective length of the head and proboscis.

Measurement of flowers, nectar volume and pollen grains

Fully opened flowers were collected and fixed immediately in 70% ethanol. The following two morphological traits of the flower were measured in the laboratory: 1) the entire length of the corolla tube and 2) the length of its tubular part (Fig. 4). Because the

corolla tube width only gradually increases distally, measuring its length is potentially arbitrary. We therefore measured the length from the basal point of the corolla to the point on the midline of the corolla at the width of 2.6mm, which is the average of the maximum width of the bee's head, as the effective length of the corolla tube.

Nectar volume of *L. gracilipes* flowers was measured by obtaining nectar from the base of the corolla tube with 0.5μl micro-capillary tubes (EM minicaps, Hirschmann Laboratories, Germany) in the morning. First, we randomly selected 70 unbagged flowers with anthers full of pollen (that is suspected to be at the onset of flowering or immediately after that), and measured their nectar volume in early April of 2011. Second, we examined daily changes in nectar production of individual flowers in the following way: at the beginning of April 2010, a total of 66 flower buds on multiple individuals were bagged; after the flowers opened, the volume of accumulated nectar was measured each morning until the flowers dropped from the plants.

Moreover, to examine the reduction in the amount of pollen per flower under open-pollination after anthesis, we carried out the following experiments in 2012 at one of the Hino populations. We collected flowers randomly from 14 individuals and stored them in 0.5ml of 70% ethanol. In the laboratory, pollen was removed from sampled anthers using an ultrasonic cleaner, and the number of grains counted under a microscope in three replicate 5.0μl drops from a single preserved sample. We then calculated the number of the remaining pollen grains per flower. The number of potential pollen grains produced by a flower was estimated from the analysis of anthers of other flowers in bud (control). The number of remaining pollen grains was compared between pollination treatments (open and control) using generalized linear mixed models (GLMMs; Poisson error distributions, log-link functions). The effects of the pollination treatments and individual plants were included as fixed and random terms, respectively. We compared this model with a null model using ANOVA.

Examination of pollination success and self-incompatibility

We examined the pollination success and self-compatibility of *L. gracilipes* by controlling pollination experiments at one of the Hino populations in 2012. Flower buds were selected randomly from 34 individual plants. We made comparisons among flowers (1) pollinated by a single visit of *A. lonicerae*; (2) the same (*A. hebes*); (3) the same (*Lasioglossum* sp., Halictidae); (4) openly pollinated (the control); (5)

cross-pollinated (hand-pollinated); and (6) self-pollinated (hand-pollinated). Except for the control, flower buds were bagged with cellophane to exclude insect visitors. The flowers of this plant are known to be self-incompatible and protandrous[15]. Two days after anthesis, the following six treatments were applied.

(1–3) Pollination after a visit by one bee: each flower was presented to a bee; after the bee's visit, the flower was bagged again. (1) *A. lonicerae*; (2) *A. hebes*; and (3) *Lasioglossum* sp.

(4) Open-pollination: flowers were not manipulated in the field.

(5) Cross-pollination: flowers were emasculated after the stigma matured; pollen grains from two or more different individuals were applied to the stigma; the flowers were bagged again.

(6) Self-pollination: flowers were hand-pollinated after anthesis with their own pollen grains before being bagged again.

These flowers were marked with tags and their mature fruits were collected a month later. The fruits were opened under a stereoscopic microscope, and seeds and ovules were categorized as 'matured', 'undeveloped' and 'unfertilized'. Undeveloped seeds were much larger than unfertilized ovules, but smaller than mature seeds. These undeveloped seeds resulted from the abortion of fertilized ovules. Fruit set was measured as the number of fruits per total flower number. Seed set was calculated as the number of seeds in a ripe fruit divided by the total number of ovules therein.

Fruit and seed set were compared among the pollination treatments using generalized linear mixed models (GLMMs) with binomial error distributions and logit-link functions, in which the effects of the pollination treatments and individual plants were included as fixed and random terms, respectively. We compared this model to a null model using ANOVA.

Molecular techniques and phylogenetic analysis

Total DNA was extracted from a hind leg of each bee preserved in 99% ethanol. The leg was ground in tubes and incubated at 55°C for 3 hours in 485μl extraction buffer containing 15μl of proteokinase K (20mg/ml). After incubation, DNA was extracted with chloroform–isoamyl-alchohol solution, precipitated with ethanol, and stored in

50µl TE buffer. The mitochondrial COI–COII region was amplified using the polymerase chain reaction (PCR) with PrimeSTAR DNA polymerase (TAKARA BIO Inc. Japan). Primers used were "Jack"[36] and "Berb" (C2-N-3661[37]). Thermal cycle parameters were as follows: 98°C for 1min; 5 cycles of denaturation at 98% for 15 sec, 45°C for 30 sec, 72°C for 20 sec; then 30 cycles at 98°C for 15 sec, 50°C for 20 sec, 72°C for 20 sec; and final extension at 72°C for 2 min. PCR products were cleaned with ExoSTAR (GE Healthcare, USA). Purified PCR products were amplified using the ABI PRISM Big Dye Terminator v3.1 Cycle Sequencing Kit (Applied Biosystems , Foster City, CA, USA). DNA sequencing was performed on an ABI PRISM 3130 Genetic Analyzer. The obtained sequences have been deposited in the DDBJ/NCBI Genbank database (Supplementary Tables S2, S3).

Sequences were aligned using MAFFT version 7.031[38] with the Auto setting. After alignment, sequence data was partitioned into three regions: COI, tRNA-Leu and COII. Before phylogenetic analysis, we checked the homogeneity of the base composition across the taxa in all regions. This is necessary because the presence of compositional heterogeneity biases phylogenetic inference[39]. Base composition of the third codon in the COII region was significantly heterogeneous across taxa ($P<0.00001$; see Supplementary Table S3). We used RY coding[40] for the third codon of COII to improve the base composition rate.

First, we performed maximum parsimony (MP) and neighbor joining (NJ) analyses using MEGA 5.1[41]. Bootstrap test was 1000 repeats at each method. For the maximum likelihood (ML) and Bayesian analyses, we removed the third codon of the COII region, because of too many substitutions for searching nucleotide substitution models. Nucleotide substitution models of the three regions were determined by the program kakusan4[42]. Protein coding regions COI and COII were partitioned by codon positions. The GTR+Gamma model was chosen using the Akaike Information Criterion (AIC) in all partitions for the ML and Bayesian analyses.

For the ML analysis, we applied the likelihood ratchets algorithm as a tree searching strategy using software RAxML, version 7.4.4[43]. Bootstrap test was 1000 repeats for the ML. We performed Bayesian analysis using MrBayes v3.1.2[44]. This analysis was conducted with two independent runs. Four chains were run for 5,000,000 generations each, and sampled every 100 generations. The first 5,000 trees were discarded as burn-in. Two independent runs were compared to assess convergence by examining

whether the average of standard deviation of split frequencies was less than 0.01. We have checked that the effective sample size of parameter values (calculated by Tracer, version 1.5) was over 100[45].

DETAILED DESCRIPTIVE RESULTS

Insect visitors to flowers of *L. gracilipes*

At least 12 insect species were observed on flowers of *L. gracilipes* over a total of 32.6 hours of observation (Table 2, Supplementary Fig. S1). Of these, females of *A. lonicerae* visited the flowers to collect both pollen and nectar. In contrast, females of *A. hebes* and two species of *Lasioglossum* visited the flowers to collect pollen only. The reason nectar is not collected by these latter three species is that their short tongues do not reach the nectar secreted from the base of the elongate narrow corolla tube (Supplementary Video S5). The most frequent visitors to the flowers were female *A. lonicerae*; the ratio of the observed number of visits of this species to the total observed number of all species was 35% (255/735 visits) (Table 2). The second and third most frequent visitors were female *A. hebes* and female *Lasioglossum* spp. and the ratios of their observed numbers to the total were 30% (218/735) and 18% (132/735), respectively. The sum of the observed number of the above four species constituted 82% of the total (Table 2).

Flower-visiting behavior

Foraging of female *A. lonicerae* on flowers of *L. gracilipes* was directed toward both nectar and pollen. At each visit to the flowers the females exhibited several behavioral sequences. The number of observations and relative frequency of each sequence are shown in Fig. 3a. From these observations it is apparent that the bees most frequently visited the flowers only to collect nectar. When they collected pollen from a flower, the bees usually obtained not only pollen but also nectar. The number of observations of both pollen and nectar collections was nearly three times that of pollen-only collections (233 vs. 86 visits). When collecting both pollen and nectar, the bees almost always collected pollen first (nectar was collected first on only two visits out of 1171 cases). This result suggests that the bees follow an innate behavioral sequence of gathering pollen first. Both “Pollen–Nectar” and “Pollen–Nectar–Pollen” sequences were sometimes difficult to distinguish, because the bees, after collecting pollen and nectar,

often rubbed the fore tarsi over the mid tarsi to transfer pollen, which appeared very similar to pollen-collecting behavior. We therefore categorized both behavioral patterns the same way. Less frequently seen was the repetition of pollen-nectar collection. The above rubbing behavior is also similar to cleaning behavior, since it is identical to the male bee's cleaning of the body and legs[46] (also in main text). Collection of purely pollen was also seen when the bees cut open flower buds with the mandibles and fore legs (Fig. 1c).

Pollen-foraging behavior

When foraging for pollen, a female hangs by her legs in an inverted position on the flower (Fig. 1b). Holding her body with the hind legs, she scrapes away pollen from the anthers by vigorous strokes of the forelegs that move alternately (Supplementary Video S2). At the same time, she often grasps and masticates the anthers or draws out pollen from them with the mandibles. Next, she rubs the fore tarsi over the mid tarsi to transfer the pollen from the fore tarsal brushes to the mid tarsal ones. Immediately after that, she passes the mid tarsi over the hind tibiae to deposit the pollen on the hind tibial scopae. Since a flower has five anthers (Supplementary Fig. S2), she turns her body towards them sequentially while collecting pollen from each anther. As a result, lumps of pollen are accumulated mainly on the hind tibiae, but also partly on the hind femora, hind trochanters, and propodeum posterolaterally. Sometimes the bee breaks flower buds with both mandibles and fore legs, forcing them open to collect pollen, nectar or both (Fig. 1c); the petals are then scored with impressions of the bee's mandibles.

Nectar-foraging behavior

When a female bee collects nectar, she inserts her head into the corolla tube, hanging inverted on the flowers with her legs. Forcing the head forward and extending the proboscis into the narrow tube, she sucks nectar concealed at the base of the tube (Fig. 1a, e). She also occasionally takes nectar after collecting pollen (Fig. 3a). During nectar extraction, she usually stops moving her legs, but occasionally continues to scrape the anthers with the fore tarsi to collect pollen.

When we observed nectar collecting in pale-colored flowers against the light, we were occasionally able to see the movement (silhouette) of the bee's proboscis. The glossa was quickly and repeatedly extended and retracted; the tip of the glossa appeared

to reach the base of the corolla (Fig. 1e2, 4; Supplementary Video S1). When the glossa was retracted, its apical portion sometimes appeared to bend as a hook.

Action of the proboscis while feeding on nectar in the laboratory

While the bee feeds on diluted honey, the entire labio-maxillary complex is fully extended anteriorly by the downward and forward swinging of the maxillary cardines (Supplementary Fig. S3; Supplementary Videos S3, S6). While the maxillae are held in a fully extended position, the labium extends and retracts repeatedly between the maxillae by rotation of the lorum (= submentum) about its distal articulations with the distal ends of the cardines (Note the location of the distal end of the lorum (arrows in Supplementary Fig. S3b) relative to the stipites). When the labium is fully extended, the glossa is protruded out of the apices of the galeae (Fig. 2a, m, Supplementary Fig. S3a2, b1); and when the labium is retracted, the glossa is also drawn under the galeae (Supplementary Fig. S3a1, b2, 3).

Extension and retraction of the proboscis while resting

The female bee is occasionally seen extending and retracting the proboscis, with the mandibles wide open, while basking in sunlight on leaves or stems of the host plant or other objects (Fig. 1d; Supplementary Video S4). When her proboscis is extended, the labio-maxillary complex is fully protracted, and the maxillae themselves are slightly spread out. When the labium is further extended between the maxillae, the glossa is looped (Fig. 1d2–3). When the labium is completely protracted, the glossa is stretched straight posterodorsally or anterodorsally (Fig. 1d4). Nectar globules (indicated n in Fig. 1d2–4) were found on the 'throat-membrane'[3] and the dorsal face of the prementum (hypopharynx). When the labio-maxillary complex is retracted by the backward-swing of the cardines, the glossa is folded double (Fig. 1d1), i.e., the main portion of the glossa is folded on the anterior (= dorsal) face of the prementum and the distal string-like portion of the glossa is folded on the posterior face of the main portion. These motions were repeated several times consecutively. We observed numerous repetitions of this behavioral sequence.

Floral morphology of *L. gracilipes*

The corolla is funnel-shaped, nodding (Supplementary Fig. S2), and usually rosy-red

but sometimes pale pink to white. Its basal half is tubular, but the remainder is gradually inflated, culminating in five apical lobes. There are five stamens arising from the corolla tube and scarcely extruding from its apex (Supplementary Fig. S2a). The style protrudes slightly to greatly beyond the apex of the corolla lobe (Supplementary Fig. S2b). The entire length of the corolla is 12.79 ± 1.34 mm (mean ± SD, n = 50) and the effective length of its tubular part is 7.98 ± 0.83 mm (n = 50) in the Hino populations (Fig. 4a), whereas in the Kyoto populations the effective length of the tubular part is 9.15 ± 0.61 mm (n = 50) and is slightly but significantly longer (t=8.03, df=98, P<0.001; Fig. 4b).

Nectar production of *L. gracilipes*

During the flowering period (4.15 ± 1.41 days (mean ± SD, n = 66) per bagged flower), nectar was produced continuously, with its volume highest in 1-day-old flowers (Fig. 3b). The nectar volume of each bagged flower per day was 0.30 ± 0.51 µl (mean ± SD, n = 246) and the total nectar volume of each flower through its flowering period was 1.11 ± 1.20 µl (mean ± SD, n = 66). Nectar volume per flower under open-pollination conditions was 0.12 ± 0.16 µl (mean ± SD, n = 50), varying greatly among flowers (Fig. 3c).

The amount of pollen remaining in flowers

The number of remaining pollen grains was much smaller in the open flowers than in the controls (open, 1288.33 ± 313.97; control, 20122.92 ± 1796.77, df = 1, X2 = 70570, P < 0.0001; Fig. 3d). This suggests that pollen is removed by insect visitors in the first couple of days of bloom and thus the majority of flowers in a blooming period have little pollen available.

Morphology and function of the head and mouthparts

Andrena lonicerae and *A. halictoides* have the following morphological characteristics of the head and labio-maxillary complex:

(1) The head is considerably elongate, averaging 1.10 times as long as broad in female *A. lonicerae* and 1.00 times as long as broad in female *A. halictoides* (Supplementary Table S4). In contrast, it averages 0.92 times as long as broad in female *A. hebes*, which also visited *L. gracilipes* flowers, but only for pollen. The ratio of the head length to the head width is significantly different among the species (Kruskal-Wallis

rank sum test of ratios: Chi^2=58.9, df=2, P<0.0001).

(2) The malar space is remarkably long (Fig. 2a, b: *c*), averaging 0.31 mm in female *A. lonicerae* and 0.26 mm in female *A. halictoides* (Supplementary Table S4). In contrast, it averages 0.11 mm in female *A. hebes*. The ratio of the malar space length to the head width is significantly different among these species, averaging 0.12 mm in female *A. lonicerae*, 0.09 mm in female *A. halictoides*, and 0.04 mm in female *A. hebes* (Supplementary Table S4).

(3) The maxillary galea is obovoid in shape (Fig. 2g, h: *f*). In contrast, it is acute at the apex (Fig. 2i: *f*) in species of other subgenera. The inner face of the galea bears a rudimentary galeal comb, comprising only one to three setulae subbasally (Fig. 2g, h: *k*). To elucidate the uniqueness of this condition, we examined the galeal comb of 17 species representative of other subgenera of *Andrena* (Supplementary Table S5): the galeal comb comprises about twenty setulae (Fig. 2i: *k*) in the other subgenera, and the rudimentary galeal comb is unique to the subgenus *Stenomelissa*.

(4) The labial glossa comprises the following three portions: (1) the basal portion that possesses the basiglossal sclerite on the anterior face (Fig. 2a: *g*; Supplementary Fig. S4c: *a*); (2) the large, rhomboid portion that extends from the basiglossal sclerite to the isthmus of the glossa (Fig. 2a: *h*); and (3) the distal, very narrow, parallel-sided portion (Fig. 2a: *i*). We refer to these as the "basal", "main", and "string-like" portions, respectively. The string-like portion is unique to *A. lonicerae* and *A. halictoides*. (The first author (A. S.) examined the holotype of *A. vitiosa* deposited at the Natural History Museum, London, UK, and found that it lacked the labio-maxillary complex.) This portion is longer than the basal and main portions taken together in *A. lonicerae* (Fig. 2a, d) but is shorter than the latter in *A. halictoides* (Fig. 2e). The paraglossa is very small and rather papilliform (compare Fig. 2d, e: *j* with Fig. 2f: *j*; Supplementary Fig. S4c: *b* with Supplementary Fig. S4b: *b*).

(5) We examined the ultrastructure of the glossa (for morphological description, see [34]). Although the basal and main portions of the glossa of *Stenomelissa* are almost the same in structure as those of the other subgenera (compare Supplementary Fig. S4c, g with Supplementary Fig. S4a, b), the string-like portion is highly specialized. The posterior face of this portion is largely occupied by the area between the seriate line (Supplementary Fig. S4g, h, l: *c*) and the bases of the seriate hairs (Supplementary

Fig. S4h, l: *d*), to which we refer as the 'seriate area' (Supplementary Fig. S4e, h, l: *e*). Its surface is arched anteriorly, is minutely and densely scaled (Fig. 2k, Supplementary Fig. S4h, i, l), and bears a median groove (Supplementary Fig. S4g, h, j–m: *f*), which is broad in the main portion (Supplementary Fig. S4g) but becomes narrow and linear in the string-like portion (Supplementary Fig. S4h) and is evanescent subapically (Fig. 2k, Supplementary Fig. S4i). The seriate hairs are very long and curved, and are densely lined along the lateral margin of the seriate area as the 'ribs' (Fig. 2l, Supplementary Fig. S4l: *d*). In the anterior face of the string-like portion, the annulate surface (Supplementary Fig. S4e, l: *i*) is much narrower than the posterior face (seriate area: Supplementary Fig. S4e, l: *e*) distally (Supplementary Fig. S4e), almost parallel-sided. Each annulus (Supplementary Fig. S4d, l: *g*)) bears several enlarged spicules (annular hairs) that are flattened, spatulate, and splayed out (Supplementary Fig. S4d, e, l: *h*). The cross section of the string-like portion is roughly T- or I-shaped, comprising the anterior and posterior parts (Supplementary Fig. S4e, l, m). The integument of the posterior part is densely packed with tissue, whereas in the anterior part there is little tissue except for a few masses of cells inside the integument, apparently containing haemolymph (Supplementary Fig. S4k, m).

Examination of pollination success and self-incompatibility

Hand-pollination experiments indicate that *L. gracilipes* is self-incompatible because it did not produce seeds after self-pollination. This is consistent with the results of Hayashibara *et al.* (2011). The results of the self-pollination treatment (invariably zero) were excluded from the following statistical comparisons of fruit and seed set among the pollination treatments, because we were not able to estimate models properly when they were included.

To evaluate potential pollen limitation, we compared fruit and seed set between open- and cross-pollinated flowers. Fruit set differs between cross- and open-pollination: flowers under cross-pollination showed lower fruit set than those examined after open-pollination (cross; 0.35, open; 0.55, df = 1, $X^2 = 18.42$, $P < 0.0001$, Supplementary Fig. S5a). In contrast, seed set did not differ between the two treatments (cross; 0.33 ± 0.04 SE, open; 0.41± 0.04 SE, df = 1, $X^2 = 0.74$, $P = 0.34$, Supplementary Fig. S5b). If *L. gracilipes* is under pollen limitation, we expect the flowers subject to

cross-pollination to produce more seeds than those subject to open-pollination. However, the seed production subject to cross-pollination was lower than that subject to open-pollination, suggesting that the emasculation treatment had a negative effect on seed production.

We compared fruit and seed set among the flowers visited by the two species of pollinators (*A. lonicerae* and *A. hebes*) and subject to open-pollination. Because the sample size of the flowers visited by *Lasioglossum* sp. was small, the results concerning this bee were excluded from the statistical comparison. We found that these did not differ among the three treatments (fruit set: *A. hebes*, 0.67; *A. lonicerae*, 0.38; open, 0.55, df = 2, $X^2 = 2.06$, $P = 0.36$; seed set: *A. hebes*, 0.44 ± 0.06 SE; *A. lonicerae*, 0.38 ± 0.05 SE; open, 0.41 ± 0.04 SE, df = 2, $X^2 = 0.20$, $P = 0.91$, Supplementary Fig. S5). This indicates that a single visitation by either of the two andrenid bees effected sufficient pollination of *L. gracilipes*.

Molecular phylogeny of *Andrena* species

Andrena lonicerae and *A. halictoides* are monophyletic sister taxa and distinct from all other Japanese and foreign *Andrena*, as evidenced by 100% bootstrap support in the ML, MP and NJ methods (for ML, Fig. 5, Supplementary Fig. S6). The Bayesian posterior probability also supported this (Bayesian posterior probability = 1.0; Supplementary Fig. S6). These two species does not form a monophyletic group with the rest of the Japanese *Andrena* species. Furthermore, *A. halictoides* is identified as ancestral and *A. lonicerae* derived, because the sequence of *A. halictoides*, compared with that of *A. lonicerae*, is more similar to those of the other *Andrena* species (Supplementary Fig. S6).

DETAILED DISCUSSION

We discuss (1) ecological relationships between the bee and the flower; (2) characteristics of the nectar- and pollen-collecting behavior of the bee; (3) uniqueness of structure of the head and mouthparts of the bee and the movement of its glossa; and (4) morphological matching between the bee's head-proboscis and the flower. We then use this evidence to infer how the morphology of the bee is adapted for collecting nectar on the flowers.

(1) Ecological relationships between the bee and the flower

A number of entomologists have pointed out special relationships between *A. lonicerae* and *L. gracilipes*. Hirashima[13] considered *A. lonicerae* (confused it with '*A. halictoides*' at that time[14]) to be monolectic (or at most oligolectic) on *L. gracilipes* because he had collected a series of its males and females on flowers of *L. gracilipes* and because its elongate head and mouthparts appeared to be suitable for drawing nectar from the tubular flowers of *L. gracilipes*. Matsumura and Munakata[47] showed that *A. halictoides* is distinctly stenotrophic on Caprifoliaceae, presenting their observation records on relative flower preference of andrenid species in Southern Hokkaido. Tadauchi and Hirashima[14] stated that *A. halictoides* visited many different flowering plants, whereas *A. lonicerae* predominantly visited the flowers of *L. gracilipes*, based on the flower records of both species. Maeta[33] and Hayashibara *et al.*[15] stated that *A. halictoides* and *A. lonicerae* are strictly oligolectic on *Weigela* and *Lonicera*, respectively, and that the elongation of the head (malar space in particular) and proboscis enables the two species to extract nectar from the elongated narrow corolla tubes of their host-plants. Hayashibara *et al.*[15] also observed some surviving females of *A. lonicerae* visit flowers of *Glechoma hederacea* L. (Lamiaceae), *Weigera hortensis* and *Abelia spathulata* Siebold et Zucc. (Caprifoliaceae), following the flowering season of *L. gracilipes* in the Sanin District of Japan. They found the bees collect pollen only from *W. hortensis*.

At our study site, we did not observe *A. lonicerae* visit other available plants (violets, cherry blossoms, etc.) during the flowering season of *L. gracilipes*, the sole exception being flowers of *Elaeagnus multiflora* var. *hortensis* (Elaeagnaceae) for nectar only (no pollen collection observed) at the end of the flowering season of *L. gracilipes* (at 10:15-11:00AM, 8 April 2013). Although we were not able to confirm that pollen and nectar of *L. gracilipes* were the sole resource for its larvae, the lack of observations of *A. lonicerae* visiting other flowering species underscores its great dependence on *L. gracilipes*.

Further, Hayashibara *et al.*[15] investigated species composition and visiting frequency of insect visitors to *L. gracilipes* flowers and regarded *A. lonicerae* as the dominant species among them, because the relative abundance of this species exceeded 70% in the total of the seven most abundant bee species. Because a portion of the body of *A. lonicerae* was smeared with pollen and touched the stigma on foraging on the flowers, they concluded that *A. lonicerae* is the sole effective pollinator of *L. gracilipes*.

In contrast, according to our data (Table 2), the relative abundance of *A. lonicerae* is only 35%, whereas that of the second most abundant visitor, *A. hebes,* is 30%. Pollination experiments also demonstrated that the pollination by *A. hebes* is as effective as that by *A. lonicerae* (Supplementary Fig. S5).

(2) Characteristics of nectar- and pollen-collecting behavior of the bee

Females of *A. lonicerae* visited flowers of *L. gracilipes* most frequently solely for nectar (Fig. 3a), possibly because each of the flowers produced only a small amount of nectar (Fig. 3b). The average nectar volume of each bagged flower per day in the Hino populations was 0.30 ± 0.51 μl (mean ± SD, n = 246). Thus the plant appears to attract as many *A. lonicerae* bees as possible to its flowers, yet avoids attracting bumblebee queens by limiting nectar volume in each blossom.

When bees collected both pollen and nectar on one visit to flowers, they almost always collected pollen first, a sequence noted by Hayashibara *et al.*[15] as well. This behavior appears adaptive because pollen, unlike nectar, is never replenished after removal from the anther by bees. Indeed, we sometimes observed that a pollen-foraging female on a flower was attacked and driven away by another female, which then started collecting pollen on the same flower.

(3) Uniqueness of the head and mouthpart anatomy of the bee and the movement of its glossa

Here we compare the head and mouthpart structure and the movement of the glossa in *A. lonicerae* and its relatives. *Andrena lonicerae* and *A. halictoides* share the peculiarity of an elongate malar space and the labium with the distal string-like portion of the glossa (Fig. 2a, b, d, e, m; Supplementary Table S4). These modifications of the head and mouthparts are unique among approximately 1,500 species of *Andrena*, hence the two species' placement in the subgenus *Stenomelissa*.

There are four species of *Andrena* in which the glossa is exceptionally elongate: *A.* (*Iomelissa*) *violae* Robertson, 1891, *A.* (*Callandrena*) *micheneriana* LaBerge, 1978, *A.* (*Stenomelissa*) *halictoides* and *A.* (*S.*) *lonicerae*. The first two species occur in the USA, and are not closely related to the other two East Asian species. *Andrena violae*, a single species in the subgenera *Iomelissa* Robertson, has a long glossa. This bee is a narrow oligolege of violets (*Viola*) and presumably the glossa is an adaptation to this flower[21,22].

Andrena micheneriana not only has an elongate glossa but its first two labial palpomeres and the galea are elongated[22]. Although the latter characteristics resemble those of the true long-tongued bees, the first two labial palpomeres are not broad or sheath-like, unlike those of the long-tongued bees.

Our detailed examination of the glossa reveals that the string-like portion bears both dense seriate hairs (Supplementary Fig. S4l: *d*) and annular hairs (Supplementary Fig. S4d, e, l: *h*). This portion is thus not only remarkably pubescent anteriorly but also has a tunnel-like space laterally (Supplementary Fig. S4l). Therefore the glossa must be efficient at retaining nectar by capillarity. When the bee foraged for nectar on the flower, the glossa was quickly and repeatedly protracted and retracted, while the maxillae was kept fully protruded (Supplementary Fig. S3a; Supplementary Video S3), as described for *A. carlini* Cockerell, 1901 by Harder[48]. When the glossa was retracted, its apical portion was sometimes bent as a hook, thus probably drawing nectar from the base of the corolla tube. As shown in Fig. 1d and Supplementary Video S4, the glossa is very flexible, which may be a consequence of the lack of dense tissue in the anterior of its string-like portion (Supplementary Fig. S4k–m). When the glossa is fully retracted between the maxillae, the nectar is probably shifted from the surface of the glossa to the anterior surface of the prementum (hypopharynx), over which the maxillary galeae (Fig. 2a: *f*) are positioned tightly together to form a tube. Through this tube, nectar must be drawn up to the mouth by the action of the ‘cibarial pump’[49]. The glossa itself has no hollow structure through which nectar passes (Supplementary Fig. S4k, m). As Snodgrass[49] pointed out for the honeybee (Supplementary Fig. S4n–q), the glossa does not serve for the intake of liquid food. On the posterior face of the glossa, there is a median groove (Supplementary Fig. S4g, h, l: *f*), through which the saliva is likely conveyed to the tip of the glossa[49].

Although the short-tongued bees generally possess a galeal comb, it is rudimentary in *Stenomelissa* (Fig. 2g, h: *k*; Supplementary Table S5), resembling in this respect the long-tongued bees, which usually lack the comb. Instead, the maxillary stipes of these bees commonly has a concavity lined with a comb of stout bristles (stipital comb) on its posterodistal margin, although the stipital comb is absent in most Megachilidae and probably in most Nomadinae; in some of those forms the concavity itself is absent[17,46]. Apparently the reduction or loss of the galeal comb has evolved independently in *Andrena* (*Stenomelissa*) and in the long-tongued bees, as suggested by the recent

phylogenetic hypotheses on Apoidea[50–52]. Such a reduction seems to be related to the formation of a long tubular proboscis in which the galeae form the roof of the food canal by being brought together around the tongue anteriorly, allowing nectar to pass through the inner wall of the canal.

Females of *A. lonicerae* sometimes extended and retracted their proboscides while resting on leaves or stems of the host plant or other sunlit objects (Fig. 1d; Supplementary Video S4). The significance of this behavior is not obvious, but it is inferred that the bees concentrate and ripen the imbibed nectar by regurgitating and exposing it on the proboscis and evaporating water from the nectar.

(4) Morphological matching between the bee's head-proboscis and the flower

Comparison of the effective lengths (EL) of the head and proboscis taken together and the corolla tube (Fig. 4) indicates that the former is a little longer than the latter in both the Hino and Kyoto populations. This coincides with the observation that the tip of the glossa reached the base of the floral tube during nectar foraging (Fig. 1e2, 4; Supplementary Video S1). Thus, almost all bees in our study populations should be able to collect nectar from the flowers (100% in the Hino and 91% in the Kyoto populations; Fig. 4).

We measured the EL of both the bee's head-proboscis and the flower, assuming the head width of bees should be equal to the width of the corolla tube opening (Fig. 4). From the EL of both the bee and the flower, we calculate the nectar-reaching rate r (reaching to the base of the flower; unit: %) as:

$$r = \frac{1}{nm}\sum_{i=0}^{n}\sum_{j=0}^{m}[1 \ \mathit{if} \ X_i \geq Y_j;\ 0 \ \mathit{if} \ X_i < Y_j]$$

where X_i and Y_j are the EL of the bee head-proboscis and corolla tube, respectively, and n and m, the sample size of the bee and flower, respectively. The reaching rate r equals 1, if all bees can reach the nectar of any flower. In the Hino populations, the reaching rate $r_H = 1$, i.e., all bees can acquire nectar from any flower. In the Kyoto populations, $r_K = 0.91$, that is, bees can reach nectar on 91% of flower-visits.

We also define the departure index d (unit: mm) and evaluate how closely the bee head and proboscis are matched to the length of the flower by comparing the EL of the

head-proboscis with that of the corolla tube, such that:

$$d = \frac{1}{n}\sum_{i=0}^{n}\left[X_i - Y_{\max}\ if\ X_i \geq Y_{\max};0\ if\ X_i \leq Y_{\max}\right]$$

The departure index of the Hino populations d_H = 0.76 mm indicates that the effective head-proboscis length of the Hino populations is less than 1 mm longer than the flower length. In the Kyoto populations, d_K = 0 mm, implying that the bee's head-proboscis length exactly matches the floral length. The EL of the bee's head-proboscis is thus finely tuned to the EL of the corolla tube in both the Hino and Kyoto populations (Fig. 4).

The elongation of the bee's head and proboscis, the ultrastructure of the glossa, especially of its string-like portion, and the movement of the glossa are here considered adaptations for extracting nectar from the tubular flower of *L. gracilipes*. Kato[28] stated that *Lonicera* is a taxon that has coevolved with bumblebees, taking as an example *L. gracilipes*, the flowers of which are visited by queens of *Bombus diversus* Smith, 1869, *Bombus hypocrita* Pérez, 1905 and *Bombus ardens* Smith, 1879. On the other hand, Hayashibara *et al.*[15] concluded that the effective pollinator of *L. gracilipes* is not bumblebees but only *A. lonicerae*; these bumblebees do not collect any pollen from the flowers nor insert their head within the corolla tubes because their proboscides are long enough to reach the nectar at the base of the flowers. In the Hino populations, we saw *B. ardens* queens visit flowers of *L. gracilipes* only five times within a total of more than 150 hours of observation. Probably the flower of *L. gracilipes* is insufficiently attractive to bumblebees because of its small amount of nectar. It is thus presumed that the pollination syndrome of *L. gracilipes* has evolved in close relation to the foraging syndrome of *A. lonicerae*, rather than that of *Bombus* spp.

Supplementary References

29. Gusenleitner, F. & Schwarz, M. Weltweite Checkliste der Bienengattung *Andrena* mit Bemerkungen und Ergänzungen zu paläarktischen Arten (Hymenoptera, Apidae, Andreninae, *Andrena*). *Entomofauna Z. Ent. Suppl.* **12,** 1–1280 (2002).

30. Kirby, W. *Monographia Apum Angliae;... Vols 1, 2.* (Ipswich, UK: privately published, 1802).

31. Chambers, V. H. Pollens collected by species of *Andrena* (Hymenoptera, Apidae). *Proc. Royal Ent. Soc. Lond. A* **43,** 155–160 (1968).

32. Cruden, R. W. Pollination biology of *Nemophila menziesii* Hydrophyllaceae with comments on the evolution of oligolectic bees. *Evolution* **26,** 373–389 (1972).

33. Maeta, Y. *Utsugi-himehanabachi (Andrena prostomias) at Gakuonji Temple in Tajima: Its Ecology and Conservation* (Kaiyu-sha, Tokyo, 2000) (In Japanese).

34. Michener, C. D. & Brooks, R. W. A comparative study of the glossae of bees (Apoidea). *Contr. Am. Ent. Inst.* **22,** i–iii, 1–73 (1984).

35. Plant, J. D. & Paulus, H. F. Comparative morphology of the postmentum of bees (Hymenoptera: Apoidea) with special remarks on the evolution of the lorum. *Z. Zool. Syst. Evol. Forsch.* **25,** 81–103 (1987).

36. Larkin, L. L., Neff, J. L. & Simpson, B. B. Phylogeny of the *Callandrena* subgenus of *Andrena* (Hymenoptera: Andrenidae) based on mitochondrial and nuclear DNA data: Polyphyly and convergent evolution. *Mol. Phylogenet. Evol.* **38,** 330–343 (2006).

37. Simon, C., Frati, F., Beckenbach, A., Crespi, B., Liu, H. & Flook, P. Evolution, weighting, and phylogenetic utility of mitochondrial gene sequences and a compilation of conserved polymerase chain reaction primers. *Ann. Ent. Soc. Am.* **87,** 651–701 (1994).

38. Katoh, K., Kuma, K., Toh, H. & Miyata, T. MAFFT version 5: improvement in accuracy of multiple sequence alignment. *Nucleic Acids Res.* **33,** 511–518 (2005).

39. Jermiin, L. S., Ho, W. Y. S., Ababneh, F., Robinson, J. & Larkum, D. W. A. The biasing effect of compositional heterogeneity on phylogenetic estimates may be underestimated. *Syst. Biol.* **53,** 638–643 (2004).

40. Woese, C. R., Achenbach, L., Rouviere, P. & Mandelco, L. Archaeal phylogeny: reexamination of the phylogenetic position of *Archaeoglobus fulgidus* in light of certain composition-induced artifacts. *Syst. Appl. Microbiol.* **14,** 364–371 (1991).

41. Tamura K., Peterson, D., Peterson, N., Stecher, G., Nei, M. & Kumar, S. MEGA5: Molecular evolutionary genetics analysis using maximum likelihood, evolutionary distance, and maximum parsimony methods. *Mol. Biol. Evol.* **28,** 2731-2739 (2011).
42. Tanabe, A. S. Kakusan: a computer program to automate the selection of a nucleotide substitution model and the configuration of a mixed model on multilocus data. *Mol. Ecol. Notes* **7,** 962-964 (2007).
43. Stamatakis, A. RAxML-VI-HPC: maximum likelihood-based phylogenetic analyses with thousands of taxa and mixed models. *Bioinformatics* **22,** 2688–2690 (2006).
44. Ronquist, F. & Huelsenbeck, J. P. MrBayes 3: Bayesian phylogenetic inference under mixed models. *Bioinformatics* **19,** 1572–1574 (2003).
45. Rambaut, A. & Drummond, A. J. Tracer v1.5. <http://beast.bio.ed.ac.uk/Tracer> (2007).
46. Jander, R. Grooming and pollen manipulation in bees (Apoidea): the nature and evolution of movements involving the foreleg. *Physiol. Entomol.* **1,** 179–194 (1976).
47. Matsumura, T. & Munakata, M. Relative abundance, phenology and flower preference of andrenid bees at Hakodateyama, Northern Japan (Hymenoptera, Apoidea). *J. Fac. Sci. Hokkaido Univ., Ser. VI, Zoology* **17,** 106–126 (1969).
48. Harder, L. D. Functional differences of the proboscides of short- and long-tongued bees (Hymenoptera, Apoidea). *Can. J. Zool.* **61,** 1580–1586 (1983).
49. Snodgrass, R. E. *Anatomy of the Honey Bee* (Comstock Publishing Associates, Ithaca, 1956).
50. Danforth, B. N., Fang, J. & Sipes, S. Analysis of family-level relationships in bees (Hymenoptera: Apiformes) using 28S and two previously unexplored nuclear genes: CAD and RNA polymerase II. *Mol. Phylogenet. Evol.* **39,** 358–372 (2006).
51. Danforth, B. N., Sipes, S., Fang, J. & Brady, S. G. The history of early bee diversification based on five genes plus morphology. *Proc. Natl. Acad. Sci. USA* **103,** 15118–15123 (2006).
52. Brady, S. G., Litman, J. R. & Danforth, B. N. Rooting phylogenies using gene duplications: An empirical example from the bees (Apoidea). *Mol. Phylogenet. Evol.* **60,** 295–304 (2010).

Legends of Supplementary Videos

Supplementary Video S1. A female of *Andrena lonicerae* taking nectar on a flower of *Lonicera gracilipes* in the field.

Supplementary Video S2. A female of *Andrena lonicerae* collecting pollen on a flower of *Lonicera gracilipes* in the field.

Supplementary Video S3. A female of *Andrena lonicerae* sucking diluted honey added to the base of the flower of *Lonicera gracilipes* that was cut for filming in the laboratory.

Supplementary Video S4. A female of *Andrena lonicerae* extending and retracting the proboscis, while basking in sunlight on a leaf of *Lonicera gracilipes*.

Supplementary Video S5. A female of *Andrena hebes* sucking diluted honey placed within the flower of *Lonicera gracilipes* that was cut for filming in the laboratory.

Supplementary Video S6. A female of *Andrena lonicerae* sucking diluted honey placed on the bottom of a film case in the laboratory.

Supplementary Table S1. The average, maximum, and minimum temparetures (°C) per season of March and April (1981–2010) in Hachioji, Tokyo (Japan Meterological Agency: http://www.data.jma.go.jp/obd/stats/etrn/view/nml_amd_10d.php?prec_no=44&block_no=0366&year=&month=&day=&view=)

	Average	Maximum	Minimum
Last ten days of March	8.7	14.1	3.6
First ten days of Apirl	11.2	16.9	5.6
Middle ten days of April	13.1	18.8	7.7

Supplementary Table S2. Voucher information and GenBank accession numbers (COI-COII) for sequences used in this study.

No.	Species	Sex	Locality	Collection date	Collector	Accession number	Reference
88	*Andrena* (*Euandrena*) *hebes*	female	Hodokubo, Hino-shi, Tokyo	14.iv.2010	AS	AB828076	Present study
88	*Andrena* (*Euandrena*) *hebes*	female	Hodokubo, Hino-shi, Tokyo	1.iv.2013	AS	AB828077	Present study
50-1	*Andrena* (*Calomelissa*) *tsukubana*	female	Inezawa, Kodama-machi, Saitama Pref.	9.vi.2009	AS	AB828085	Present study
50-2	*Andrena* (*Calomelissa*) *tsukubana*	female	Inezawa, Kodama-machi, Saitama Pref.	9.vi.2009	AS	AB828086	Present study
51	*Andrena* (*Calomelissa*) *prostomias*	female	Inezawa, Kodama-machi, Saitama Pref.	9.vi.2009	AS	AB828078	Present study
51	*Andrena* (*Calomelissa*) *prostomias*	female	Inezawa, Kodama-machi, Saitama Pref.	9.vi.2009	AS	AB828079	Present study
60	*Andrena* (*Stenomelissa*) *halictoides*	female	Naeba, Yuzawa-machi, Niigata Pref.	16.vi.2009	AS	AB828089	Present study
60	*Andrena* (*Stenomelissa*) *halictoides*	female	Naeba, Yuzawa-machi, Niigata Pref.	16.vi.2009	AS	AB828090	Present study
61-1	*Andrena* (*Stenomelissa*) *lonicerae*	female	Tenjô-yama, Kawaguchi-ko, Yamanashi Pref.	15.v.2009	HT	AB828073	Present study
61-1	*Andrena* (*Stenomelissa*) *lonicerae*	male	Hodokubo, Hino-shi, Tokyo	30.iii.2010	AS	AB828075	Present study
61-2	*Andrena* (*Stenomelissa*) *lonicerae*	female	Hodokubo, Hino-shi, Tokyo	9.v.2009	HT	AB828074	Present study
91	*Andrena* (*Chlorandrena*) *knuthi*	female	Kanasana-jinja, Kamikawa-machi, Saitama Pref.	10.vi.2009	AS	AB828080	Present study
91	*Andrena* (*Chlorandrena*) *knuthi*	female	Kanasana-jinja, Kamikawa-machi, Saitama Pref.	10.vi.2009	AS	AB828081	Present study
91	*Andrena* (*Chlorandrena*) *knuthi*	male	Naeba, Yuzawa-machi, Niigata Pref.	16.vi.2009	AS	AB828082	Present study
57	*Andrena* (*Plastandrena*) *japonica*	male	Naeba, Yuzawa-machi, Niigata Pref.	16.vi.2009	AS	AB828083	Present study
93-1	*Andrena* (*Simandrena*) *kerriae*	female	Koke-zawa, Takao-san, Tokyo	24.v.2009	AS	AB828084	Present study
93-1	*Andrena* (*Simandrena*) *nippon*	female	Yarimizu, Hachioji-shi, Tokyo	20.v.2009	AS	AB828087	Present study
93-2	*Andrena* (*Simandrena*) *opacifovea*	female	Yarimizu, Hachioji-shi, Tokyo	20.v.2009	AS	AB828091	Present study
80	*Andrena* (*Andrena*) *ishiharai*	female	Koke-zawa, Takao-san, Tokyo	23.v.2009	AS	AB828092	Present study
80	*Andrena* (*Andrena*) *ishiharai*	male	Koke-zawa, Takao-san, Tokyo	23.v.2009	AS	AB828093	Present study
79	*Andrena* (*Melandrena*) *watasei*	male	Senga-taki, Karuizawa-machi, Nagano Pref.	15.vi.2009	AS	AB828088	Present study
9-2	*Andrena* (*Callandrena*) *accepta*					AF504313	1
9-1	*Andrena* (*Callandrena*) *accepta*					AF504314	1
24	*Andrena* (*Callandrena*) *afimbriata*					AF504315	1
71	*Andrena* (*Callandrena*) *aliciae*					AF504316	1
7	*Andrena* (*Callandrena*) *aliciarum*					AF504317	1
35	*Andrena* (*Callandrena*) *ardis*					AF504318	1
34	*Andrena* (*Callandrena*) *asteris*					AF504319	1
18	*Andrena* (*Callandrena*) *auripes*					AF504320	1
68	*Andrena* (*Callandrena*) *barberi*					AF504321	1
38	*Andrena* (*Callandrena*) *beameri*					AF504322	1
37	*Andrena* (*Callandrena*) *biscutellata*					AF504323	1
69	*Andrena* (*Callandrena*) *braccata*					AF504324	1
33	*Andrena* (*Callandrena*) *bullata*					AF504325	1
14-2	*Andrena* (*Callandrena*) *calvata*					AF504326	1
14-1	*Andrena* (*Callandrena*) *calvata*					AF504327	1
27	*Andrena* (*Callandrena*) *crawfordii*					AF504328	1
64	*Andrena* (*Callandrena*) *discreta*					AF504329	1
30	*Andrena* (*Callandrena*) *fulvipennis*					AF504330	1
65	*Andrena* (*Callandrena*) *fumosa*					AF504331	1
39	*Andrena* (*Callandrena*) *gardineri*					AF504332	1
22	*Andrena* (*Callandrena*) *haynesi*					AF504333	1
72	*Andrena* (*Callandrena*) *helianthi*					AF504334	1
72	*Andrena* (*Callandrena*) *helianthi*					AF504335	1
38	*Andrena* (*Callandrena*) *helianthiformis*					AF504336	1
8	*Andrena* (*Callandrena*) *humeralis*					AF504337	2
16	*Andrena* (*Callandrena*) *inculta*					AF504338	1
28	*Andrena* (*Callandrena*) *krigiana*					AF504340	1
29	*Andrena* (*Callandrena*) *krigiana*					AF504341	1
6	*Andrena* (*Callandrena*) *levigata*					AF504342	1
42-2	*Andrena* (*Callandrena*) *melliventris*					AF504344	1
42-1	*Andrena* (*Callandrena*) *melliventris*					AF504345	1
10	*Andrena* (*Callandrena*) *micheneriana*					AF504346	1
101-1	*Andrena* (*Callandrena*) *ofella*					AF504348	1
101-2	*Andrena* (*Callandrena*) *ofella*					AF504349	1
67	*Andrena* (*Callandrena*) *pecosana*					AF504350	1
66	*Andrena* (*Callandrena*) *pecosana*					AF504351	1
13	*Andrena* (*Callandrena*) *perpunctata*					AF504352	1
31	*Andrena* (*Callandrena*) *placata*					AF504353	1
11	*Andrena* (*Callandrena*) *reflexa*					AF504354	1
15	*Andrena* (*Callandrena*) *rubens*					AF504355	1
41	*Andrena* (*Callandrena*) *rudbeckiae*					AF504356	1
26	*Andrena* (*Callandrena*) *senticulosa*					AF504357	1
32-3	*Andrena* (*Callandrena*) *simplex*					AF504358	1
32-1	*Andrena* (*Callandrena*) *simplex*					AF504359	1
32-2	*Andrena* (*Callandrena*) *simplex*					AF504360	1
17	*Andrena* (*Callandrena*) *simulata*					AF504361	1
23-1	*Andrena* (*Callandrena*) *sitiliae*					AF504362	2
23-2	*Andrena* (*Callandrena*) *sitiliae*					AF504363	2
19	*Andrena* (*Callandrena*) *tegularis*					AF504364	1
25	*Andrena* (*Callandrena*) *tonkaworum*					AF504365	1
40	*Andrena* (*Callandrena*) *utahensis*					AF504366	1
21	*Andrena* (*Callandrena*) *verecunda*					AF504367	1
21	*Andrena* (*Callandrena*) *verecunda*					AF504368	1
12	*Andrena* (*Callandrena*) *brooksi*					AF504370	1
36	*Andrena* (*Callandrena*) *chapparalensis*					AF504371	1
100	*Andrena* (*Callandrena*) *neffi*					AF504372	1
63	*Andrena* (*Callandrena*) *vogleri*					AF504373	1
20	*Andrena* (*Callandrena*) aff. *manifesta*					AF504374	1
70	*Andrena* (*Callandrena*) aff. *rava*					AF504375	1
84	*Andrena* (*Andrena*) *macoupinensis*					AF504377	1
85	*Andrena* (*Archiandrena*) *banksi*					AF504378	1
98	*Andrena* (*Belandrena*) *sagittagalea*					AF504379	1
83	*Andrena* (*Cnemidandrena*)*citrinihirta*					AF504380	1
82	*Andrena* (*Cnemidandrena*) *nubecula*					AF504381	1
81	*Andrena* (*Cnemidandrena*) n. sp.48					AF504382	1
56	*Andrena* (*Diandrena*) *anatolis*					AF504383	1
55	*Andrena* (*Diandrena*) n. sp.120					AF504384	1
89	*Andrena* (*Euandrena*) *geranii*					AF504385	1
43	*Andrena* (*Gonandrena*) *avulsa*					AF504386	1
62	*Andrena* (*Gonandrena*) *nigrifrons*					AF504387	2
87	*Andrena* (*Holandrena*) *cressonii*					AF504388	1
95	*Andrena* (*Larandrena*) *miserabilis*					AF504389	1
76	*Andrena* (*Leucandrena*) *faceta*					AF504390	1
78	*Andrena* (*Melandrena*) *carlini*					AF504391	1
73	*Andrena* (*Melandrena*) *dolomellea*					AF504392	1
74	*Andrena* (*Melandrena*) *nivalis*					AF504393	1
75	*Andrena* (*Melandrena*) *vicina*					AF504394	1
102	*Andrena* (*Micrandrena*) *illinoiensis*					AF504395	2
54	*Andrena* (*Onagrandrena*) *linsleyi*					AF504396	1

99-1	*Andrena* (*Parandrena*) *andrenoides*	AF504397	1
99-2	*Andrena* (*Parandrena*) *arenicola*	AF504398	1
59	*Andrena* (*Plastandrena*) *crataegi*	AF504399	1
58	*Andrena* (*Plastandrena*) *mellea*	AF504400	1
90	*Andrena* (*Ptilandrena*) *erigeniae*	AF504401	1
62	*Andrena* (*Poecilandrena*) *viciae*	AF504402	2
49	*Andrena* (*Rhaphandrena*) *dapsilis*	AF504404	1
103	*Andrena* (*Scaphandrena*) *primulifrons*	AF504405	1
104	*Andrena* (*Scaphandrena*) *trapezoidea*	AF504406	1
86	*Andrena* (*Scaphandrena?*) n. sp.78	AF504407	1
52	*Andrena* (*Scrapteropsis*) *flaminea*	AF504408	1
48	*Andrena* (*Scrapteropsis*) *ilicis*	AF504409	1
44	*Andrena* (*Scrapteropsis*) *imitatrix*	AF504410	1
53	*Andrena* (*Scrapteropsis*) *unicostata*	AF504411	1
92	*Andrena* (*Simandrena*) *nasonii*	AF504412	1
94	*Andrena* (*Taeniandrena*) *wilkella*	AF504413	1
47	*Andrena* (*Trachandrena*) *forbesii*	AF504414	1
45	*Andrena* (*Trachandrena*) *miranda*	AF504415	1
46	*Andrena* (*Trachandrena*) *nuda*	AF504416	1
77	*Andrena* (*Tylandrena*) *perplexa*	AF504417	1
4	*Anthrenoides* n. sp.	AF504419	2
52???	*Macrotera texana*	n.a.	1
1	*Protandrena* (*Heterosarus*) *albitarsus*	AF504422	1
3	*Protandrena* (*Protandrena*) *bancrofti*	AF504423	1
5	*Psaenythia* sp.	AF504424	1
2	*Pseudopanurgus rugosus*	AF504425	1
15	*Andrena* (*Callandrena*) aff. *rubens*	AF506836	2
96-1	*Andrena* (*Hoplandrena*) *rosae*	EU374680	3
96-1	*Andrena* (*Hoplandrena*) *rosae*	EU374681	3
96-1	*Andrena* (*Hoplandrena*) *rosae*	EU374682	3
96-2	*Andrena* (*Hoplandrena*) *rosae*	EU374683	3
96-1	*Andrena* (*Hoplandrena*) *rosae*	EU374684	3
96-1	*Andrena* (*Hoplandrena*) *rosae*	EU374685	3
97-1	*Andrena* (*Hoplandrena*) *carantonica*	EU374686	3
97-1	*Andrena* (*Hoplandrena*) *carantonica*	EU374687	3
97-2	*Andrena* (*Hoplandrena*) *carantonica*	EU374688	3
97-2	*Andrena* (*Hoplandrena*) *carantonica*	EU374689	3

Collector's abbreviation: AS, A. Shimizu; HT, H. Takahashi. Reference: 1, Larkin *et al*. 2005; 2, Larkin *et al*. 2006; 3, Reemer *et al*., unpublished. Vouchers of the Japanese species are deposited at the Insect Collection of the Laboratory of Systematic Zoology, Tokyo Metropolitan University.

Supplementary Table S3. The regions used for molecular phylogenetic analysis of *Andrena*.

Data partition	Characters	A (%)	C (%)	G (%)	T (%)	CI, RI	P
COI partial region	42	39.2	17.8	4.7	38.2	0.35, 0.74	
COI first codon	14	46.2	14.8	12.1	27		1.0000
COI second codon	14	35.1	18.5	0.5	45.9		1.0000
COI third codon	14	36.4	20.2	1.6	41.7		0.9177
tRNA-Leu	55	39.9	11.9	14.2	34.0	0.40, 0.8	1.0000
COII partial region	558	41	19.2	8.1	31.6	0.14, 0.58	
COII first codon	186	45.9	16.5	14.5	23.1		1.0000
COII second codon	186	27.8	23.7	9	39.5		1.0000
COII third codon	186	49.4	17.4	1	32.2		< 0.0001
COII third codon (RY coding)	186						1.0000
Combine (with COII third codon; RY coding)	655					0.25, 0.61	1.0000
Combine (without COII third codon)	655					0.25, 0.61	1.0000

Supplementary Table S4. Measurements (mm) of female head length (HL), head width (HW), malar space length (MSL), proboscis length (PL) and effective length of the head and proboscis* (HL+PL) and ratios of HL/HW and MSL/HW of three *Andrena* species. Figures in parentheses indicate sample size.

	A. lonicerae	*A. halictoides*	*A. hebes*
HL (mm)	2.9 ± 0.07 (31)	2.9 ± 0.07 (11)	2.6 ± 0.07 (28)
HW (mm)	2.6 ± 0.05 (31)	2.9 ± 0.09 (11)	2.8 ± 0.08 (28)
MSL (mm)	0.3 ± 0.02 (31)	0.3 ± 0.01 (11)	0.1 ± 0.01 (28)
PL (mm)	5.7 ± 0.14 (27)	4.3 ± 0.14 (10)	2.6 ± 0.08 (17)
HL+PL (mm)	10.6 ± 0.21 (17)		5.0, 5.5
HL/HW	1.10 ± 0.02 (31)	0.99 ± 0.02 (11)	0.92 ± 0.02 (28)
MSL/HW	0.12 ± 0.01 (31)	0.09 ± 0.00 (11)	0.04 ± 0.00 (28)

*Length from the glossal tip to the point on the midline of the head at the maximum head width. Variation among the four traits, HL, HW, MSL and PL tested using MANOVA: $F(8,96)=273.03$, $P<0.0001$. Individual ANOVAs showed significant variation ($P<0.001$) in all structures. Post hoc Tukey test showed that all pairwise comparisons were significant. Variation among the ratios was tested using the Kruskal-Wallis rank sum test and a oneway ANOVA using log-transformed data. HL/HW: Chi-square=58.9, df=2, $P<0.0001$. MSL/HW: Chi-square=58.7, df=2, $P<0.0001$. HL/HW: $F(2,67)=609.0$, $P<0.0001$. MSL/HW: $F(2,67) =3356.4$, $P<0.0001$. Post hoc Tukey test showed that all pairwise comparisons were significant.

Supplementary Table S5. Condition of the galeal comb in species of *Andrena* and *Panurginus* (Andrenidae).

Species	Galeal comb
Andrena (*Andrena) hondoica*	complete
A. (*Calomelissa*) *tsukubana*	complete
A. (*Chlorandrena) knuthi*	complete
A. (*Cnemiandrena) denticulata seneciorum*	complete
A. (*Euandrena) hebes*	complete
A. (*Gymnandrena*) *parathoracica*	complete
A. (*Habromelissa) omogensis*	complete
A. (*Holandrena*) *valeriana*	complete
A. (*Hoplandrena) dentata*	complete
A. (*Leucandrena) richardsi*	complete
A. (*Micrandrena*) *semirugosa brassicae*	complete
A. (*Notandrena) nitidiuscula*	complete
A. (*Oreomelissa*) *mitakensis*	complete
A. (*Plastandrena*) *japonica*	complete
A. (*Simandrena*) *opacifovea*	complete
A. (*Stenomelissa) halictoides*	rudimentary
A. (*Stenomelissa*) *lonicerae*	rudimentary
A. (*Taeniandrena) ezoensis*	complete
Panurginus (*Panurginus*) *crawfordi*	complete

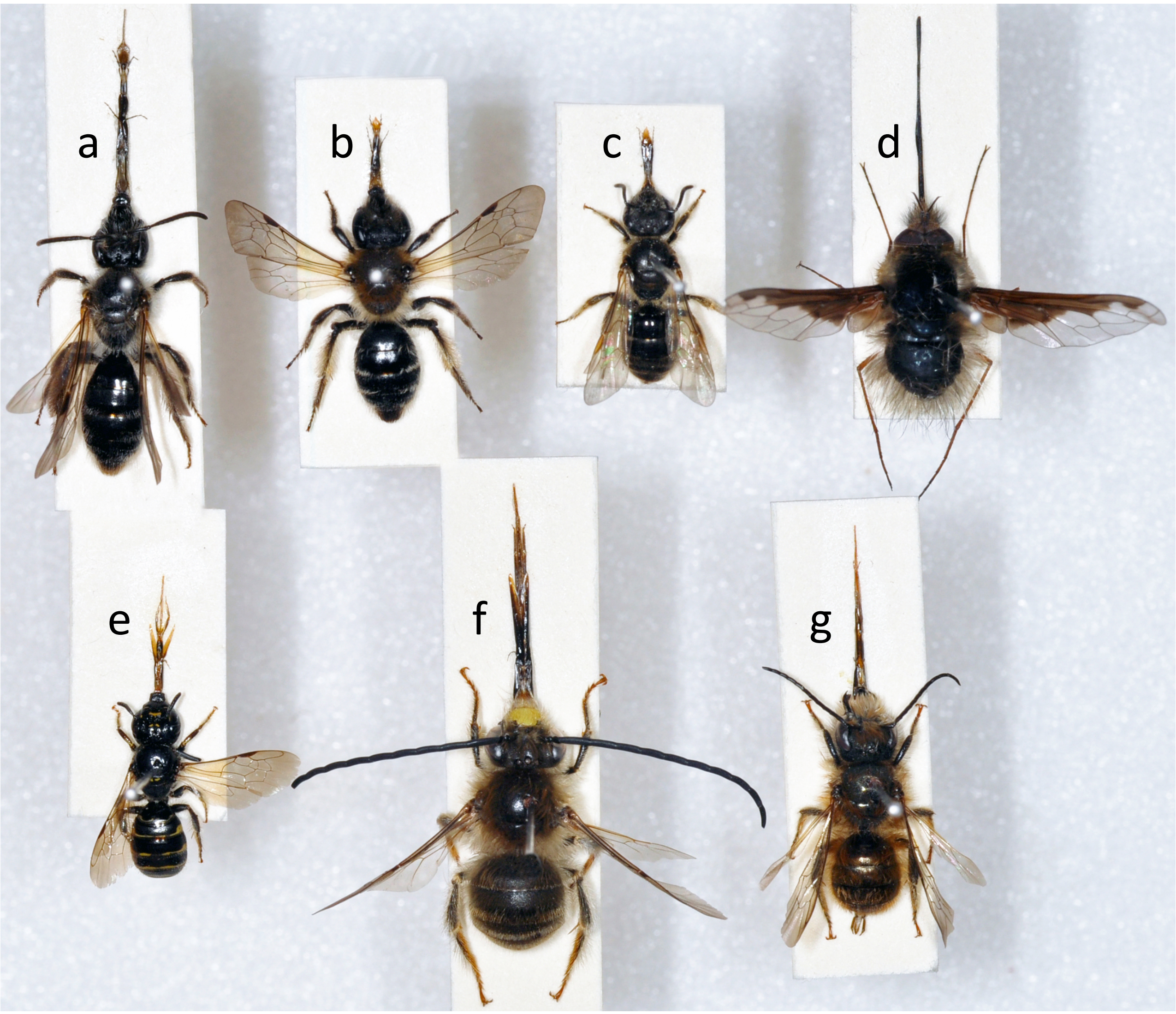

Supplementary Fig. S1. Frequent insect visitors to flowers of *Lonicera gracilipes* at the Hino study site (see also Table 2). a, *Andrena* (*Stenomelissa*) *lonicerae* Tadauchi & Hirashima, 1989, female (Andrenidae); b, *A.* (*Euandrena*) *hebes* Pérez, 1905, female (Andrenidae); c, *Lasioglossum* (*Evylaeus*) sp., female (Halictidae); d, *Bombylius major* Linnaeus, 1758, male (Bombyliidae); e, *Ceratina japonica* Cockerell, 1911, female (Anthophoridae); f, *Eucera nipponensis* (Pérez, 1911), male (Anthophoridae); g, *Osmia taurus* Smith, 1873, male (Megachilidae). Note that a–c are short-tongued bees, d is a bee fly, and e–g are long-tongued bees. (Photographed by A. Shimuzu.)

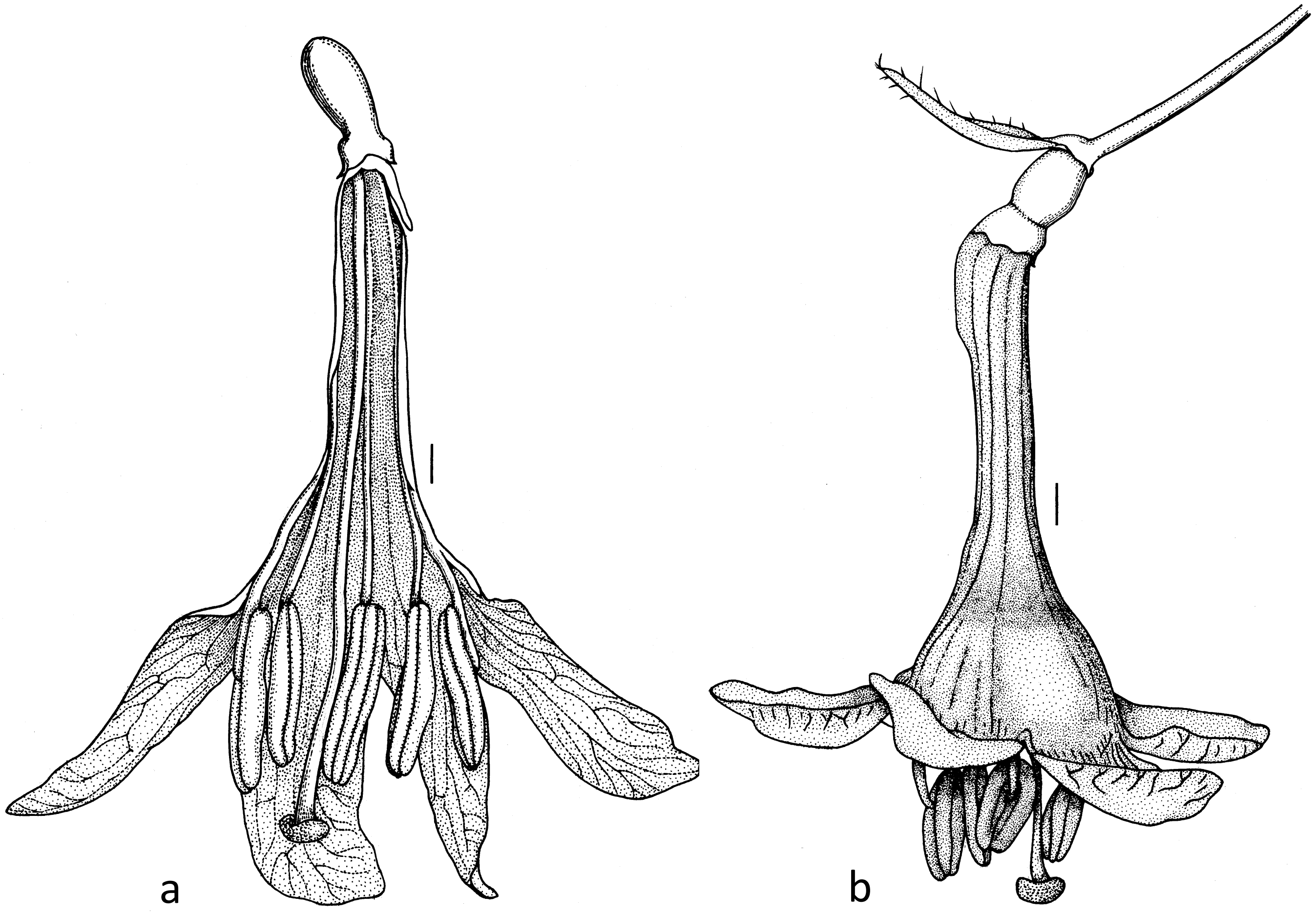

Supplementary Fig. S2. The flower of *Lonicera gracilipes*. (a) Cross section. (b) Overview. (Drawn by A. Shimuzu.)

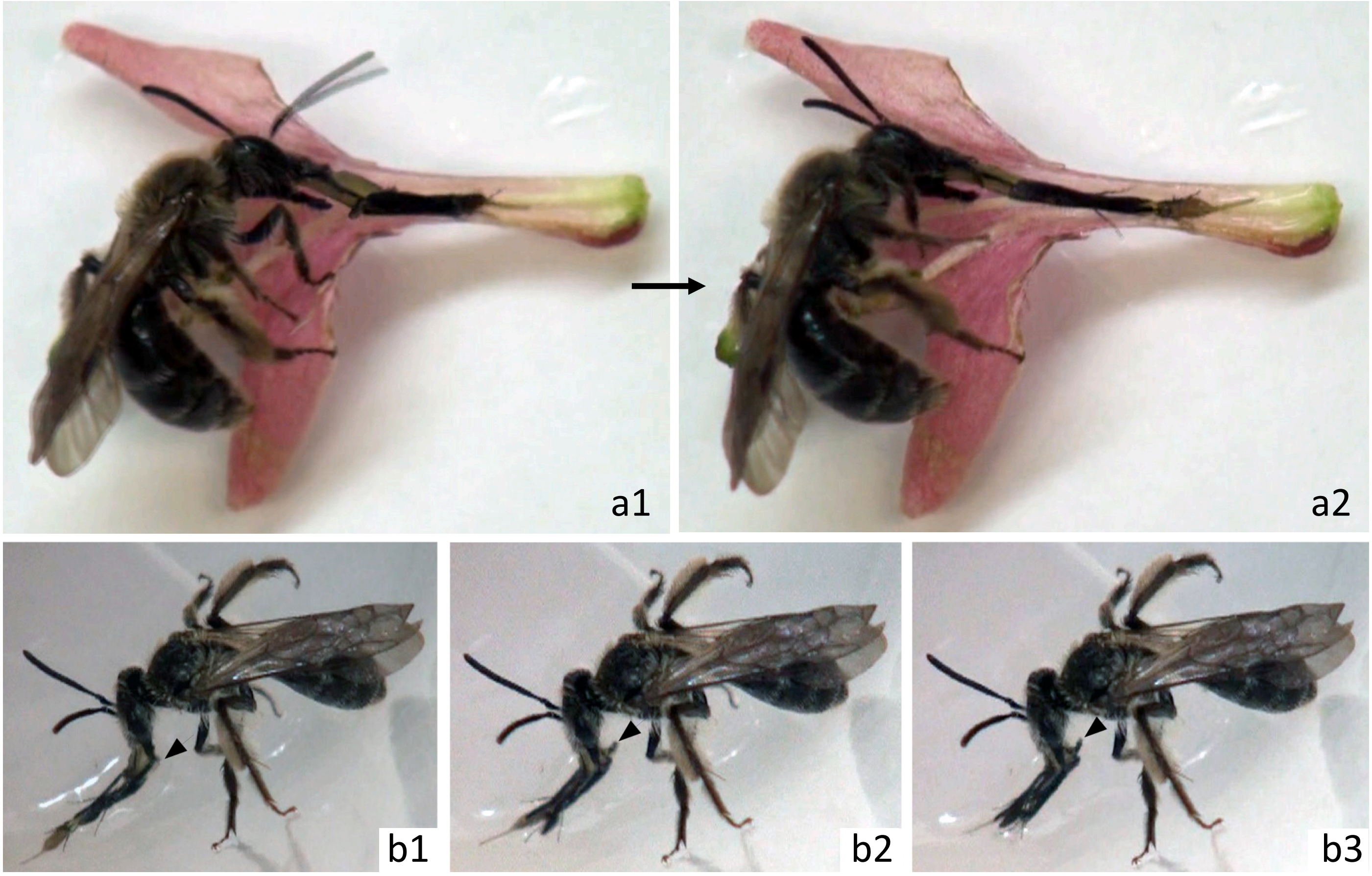

Supplementary Fig. S3. Video pictures of honey sucking of a female *Andrena lonicerae* in the laboratory. (a) Diluted honey was added to the base of a *Lonicera gracilipes* flower that was cut for filming. (b) Diluted honey was placed on the bottom of a film case. Arrow, the distal end of the lorum. (Photographed by A. Shimuzu.)

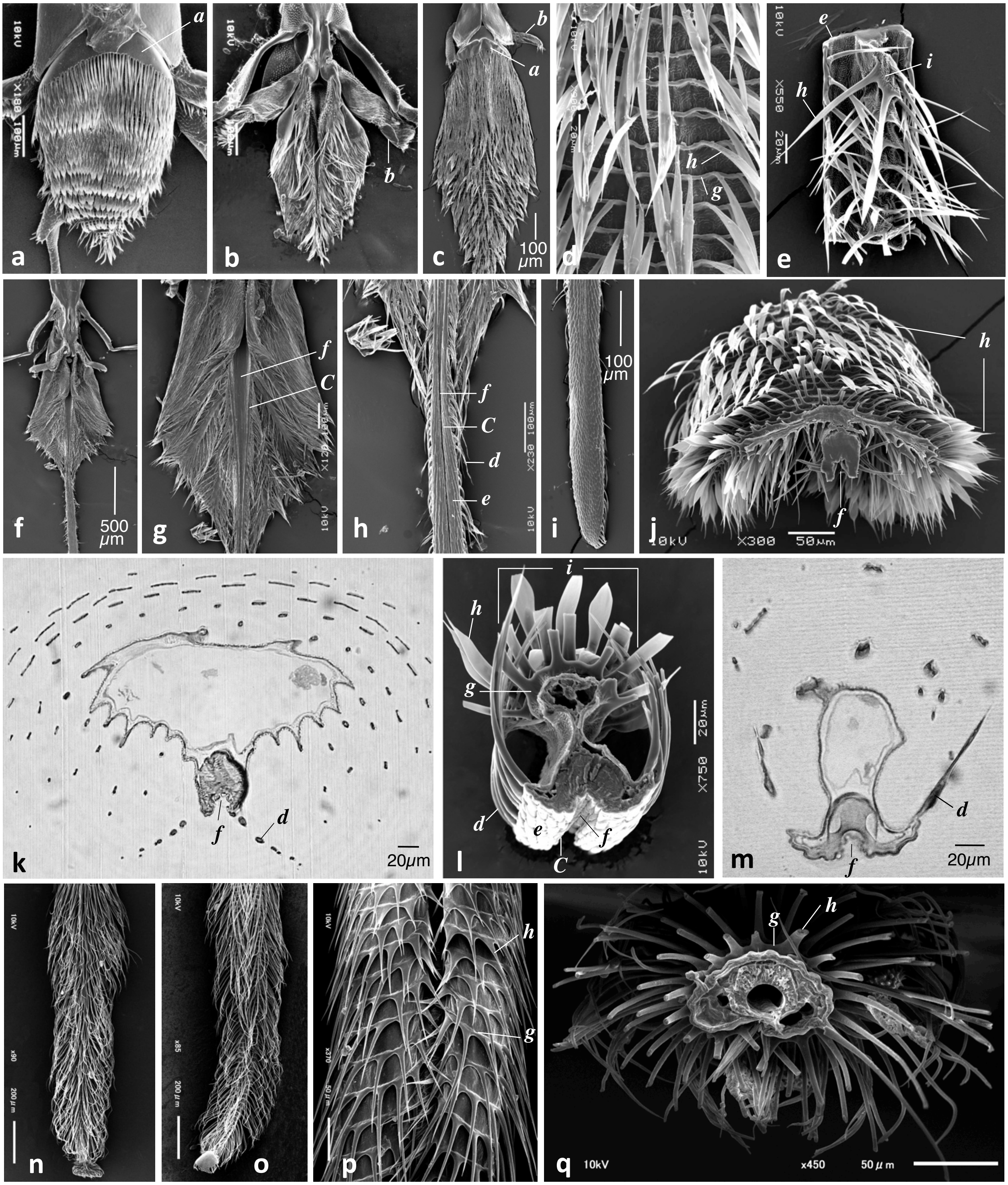
a
b
c
d
e
f
g
h
i
j
k
l
m
n
o
p
q
100 μm
500 μm
100 μm
10kV X300 50μm
20μm
20μm
X750 20μm
10kV
x450
50 μ m

Supplementary Fig. S4. Ultrastructure of female andrenid glossae, observed with an optical microscope (k, m) or scanned by SEM (others). a–b, *Andrena hebes*: (a) the whole glossa, anterior view; (b) same, posterior view. c–m, *A. lonicerae*: (c) basal and main portions, anterior view; (d) annulate surface (anterior view); (e) part of string-like portion, anterolateral view; (f) basal and main portions and part of string-like portion, posterior view; (g) main portion, posterior view; (h) basal part of string-like portion, posterior view; (i) distal part of string-like portion, posterior view; (j) cross section of main portion, anterior face up; (k) same; (l) cross section of string-like portion, anterior face up; and (m) same. N–Q, *Apis mellifera* Linnaeus, 1758: (n) distal part of glossa, anterior view; (o) same, posterior view; (p) middle part of glossa enlarged, posterior view; (q) cross section of glossa, anterior face up. *a*, basiglossal sclerite of glossa; *b*, paraglossa; *c*, seriate line; *d*, seriate hair; *e*, seriate area; *f*, median groove; *g*, annulus; *h*, annular hair; *i*, annulate surface. (Photographed by A. Shimuzu.)

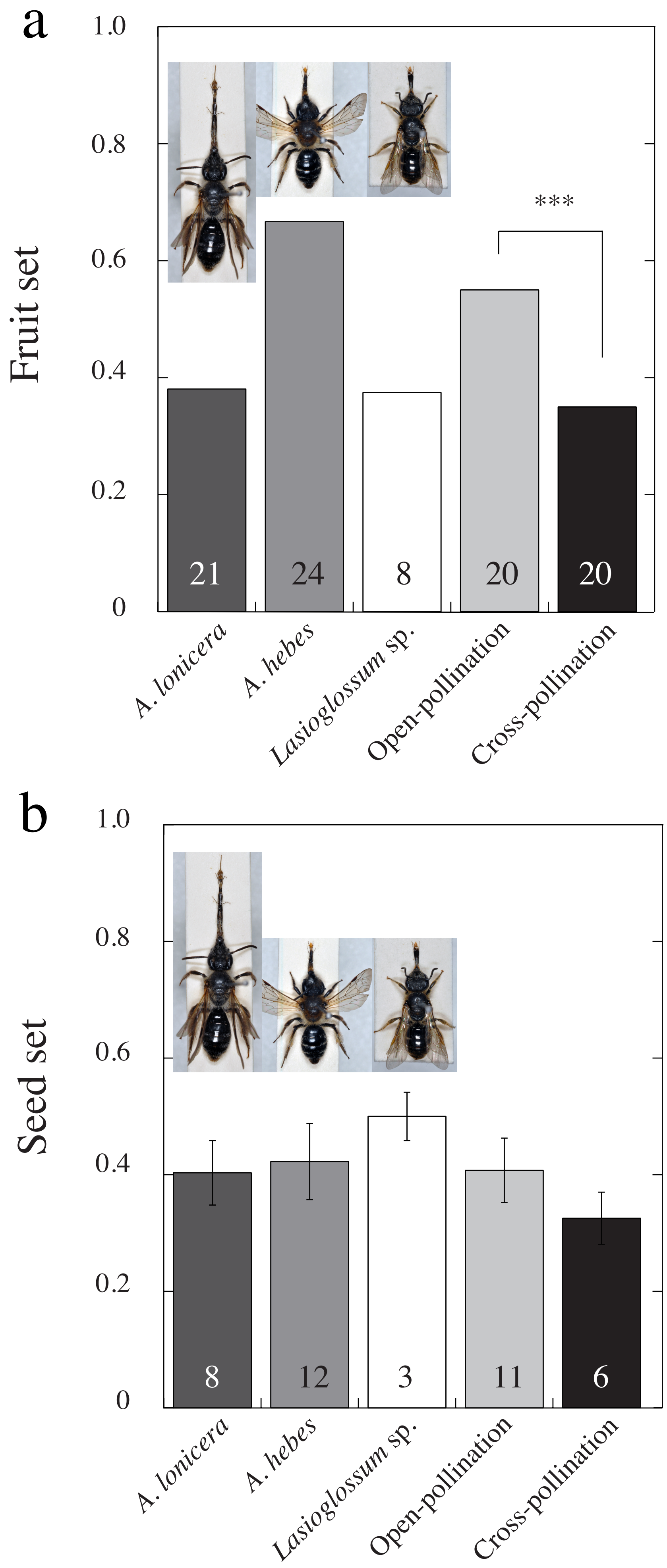
a
1.0
0.8
0.6
0.4
0.2
0
Fruit set
***
21
24
8
20
20
A. lonicera
A. hebes
Lasioglossum sp.
Open-pollination
Cross-pollination
b
1.0
0.8
0.6
0.4
0.2
0
Seed set
8
12
3
11
6
A. lonicera
A. hebes
Lasioglossum sp.
Open-pollination
Cross-pollination

Supplementary Fig. S5. Seed production after a single visitation by the two andrenid species (*Andrena lonicerae* and *A. hebes*) and a halictid species (*Lasioglossum* sp.) and by experimental pollination. (a) Fruit set and (b) seed set (Mean ± SE). Numbers within bars represent sample sizes. Fruit and seed set were compared between open- and cross-pollinated flowers. Fruit set of cross-pollinated flowers was significantly lower than that of open-pollinated flowers (*** $P < 0.0001$ from the GLMMs and ANOVA). Fruit and seed set after a single visitation of *A. lonicerae* and *A. hebes* were compared with those in open-pollination. There were no significant differences among the three treatments. Because the sample size of the flowers visited by *Lasioglossum* sp. was small, the results concerning this bee were excluded from the statistical comparison.

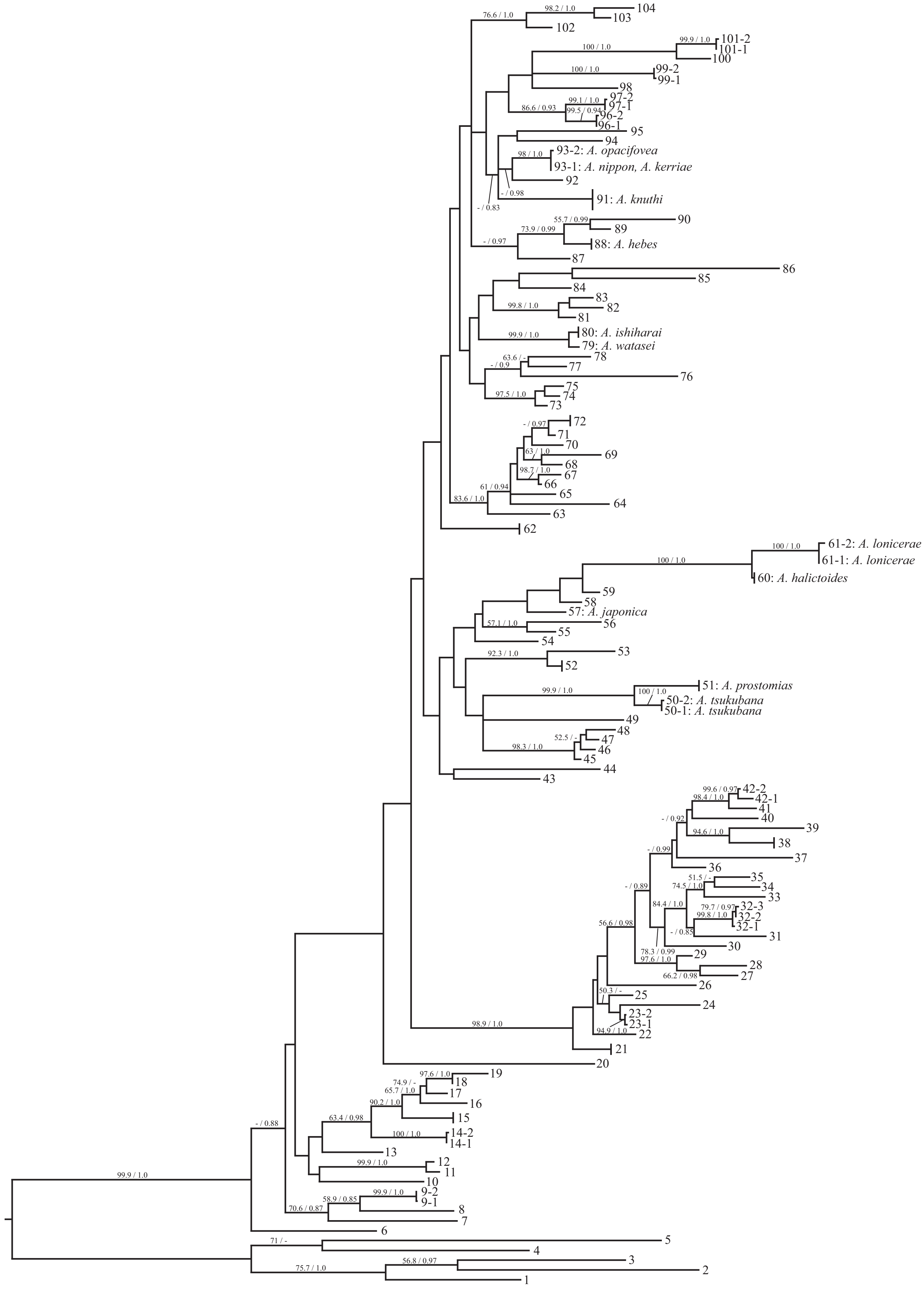

76.6 / 1.0
98.2 / 1.0
104
103
102
100 / 1.0
99.9 / 1.0
101-2
101-1
100
100 / 1.0
99-2
99-1
98
99.1 / 1.0
97-2
97-1
86.6 / 0.93
99.5 / 0.94
96-2
96-1
95
94
98 / 1.0
93-2: *A. opacifovea*
93-1: *A. nippon*, *A. kerriae*
92
- / 0.98
91: *A. knuthi*
- / 0.83
55.7 / 0.99
90
89
73.9 / 0.99
88: *A. hebes*
- / 0.97
87
86
85
84
83
82
99.8 / 1.0
81
99.9 / 1.0
80: *A. ishiharai*
79: *A. watasei*
63.6 / -
78
- / 0.9
77
76
75
74
97.5 / 1.0
73
- / 0.97
72
71
70
63 / 1.0
69
68
98.7 / 1.0
67
66
61 / 0.94
65
64
83.6 / 1.0
63
62
100 / 1.0
61-2: *A. lonicerae*
100 / 1.0
61-1: *A. lonicerae*
60: *A. halictoides*
59
58
57: *A. japonica*
57.1 / 1.0
56
55
54
92.3 / 1.0
53
52
51: *A. prostomias*
99.9 / 1.0
100 / 1.0
50-2: *A. tsukubana*
50-1: *A. tsukubana*
49
48
52.5 / -
47
46
98.3 / 1.0
45
44
43
99.6 / 0.97
42-2
98.4 / 1.0
42-1
41
40
- / 0.92
39
94.6 / 1.0
38
37
- / 0.99
36
51.5 / -
35
74.5 / 1.0
34
- / 0.89
33
84.4 / 1.0
79.7 / 0.97
32-3
99.8 / 1.0
32-2
32-1
- / 0.85
31
56.6 / 0.98
30
78.3 / 0.99
29
97.6 / 1.0
28
66.2 / 0.98
27
26
50.3 / -
25
24
23-2
23-1
94.9 / 1.0
22
98.9 / 1.0
21
20
97.6 / 1.0
19
74.9 / -
18
65.7 / 1.0
17
90.2 / 1.0
16
15
63.4 / 0.98
100 / 1.0
14-2
14-1
13
99.9 / 1.0
12
11
10
- / 0.88
99.9 / 1.0
99.9 / 1.0
9-2
58.9 / 0.85
9-1
70.6 / 0.87
8
7
6
71 / -
5
4
3
56.8 / 0.97
75.7 / 1.0
2
1
0.3

Supplementary Fig. S6. The maximum likelihood tree of the genus *Andrena* based on the mitochondrial COI, tRNA-Leu and COII regions. Numbers above branches indicate bootstrap support of the ML and the Bayesian posterior probabilities. The operational taxonomic units (species) except the Japanese species were indicated in numbers. Species names were shown in Supplementary Table S2.
